# A fruit quality gene map of *Prunus*

**DOI:** 10.1186/1471-2164-10-587

**Published:** 2009-12-08

**Authors:** Ebenezer A Ogundiwin, Cameron P Peace, Thomas M Gradziel, Dan E Parfitt, Fredrick A Bliss, Carlos H Crisosto

**Affiliations:** 1 Plant Sciences Department, University of California Davis, 1 Shields Ave., Davis CA 95616, USA; 2 Department of Horticulture and Landscape Architecture, Washington State University, Pullman, WA 99164 USA

## Abstract

**Background:**

*Prunus *fruit development, growth, ripening, and senescence includes major biochemical and sensory changes in texture, color, and flavor. The genetic dissection of these complex processes has important applications in crop improvement, to facilitate maximizing and maintaining stone fruit quality from production and processing through to marketing and consumption. Here we present an integrated fruit quality gene map of *Prunus *containing 133 genes putatively involved in the determination of fruit texture, pigmentation, flavor, and chilling injury resistance.

**Results:**

A genetic linkage map of 211 markers was constructed for an intraspecific peach (*Prunus persica*) progeny population, Pop-DG, derived from a canning peach cultivar 'Dr. Davis' and a fresh market cultivar 'Georgia Belle'. The Pop-DG map covered 818 cM of the peach genome and included three morphological markers, 11 ripening candidate genes, 13 cold-responsive genes, 21 novel EST-SSRs from the ChillPeach database, 58 previously reported SSRs, 40 RAFs, 23 SRAPs, 14 IMAs, and 28 accessory markers from candidate gene amplification. The Pop-DG map was co-linear with the *Prunus *reference T × E map, with 39 SSR markers in common to align the maps. A further 158 markers were bin-mapped to the reference map: 59 ripening candidate genes, 50 cold-responsive genes, and 50 novel EST-SSRs from ChillPeach, with deduced locations in Pop-DG via comparative mapping. Several candidate genes and EST-SSRs co-located with previously reported major trait loci and quantitative trait loci for chilling injury symptoms in Pop-DG.

**Conclusion:**

The candidate gene approach combined with bin-mapping and availability of a community-recognized reference genetic map provides an efficient means of locating genes of interest in a target genome. We highlight the co-localization of fruit quality candidate genes with previously reported fruit quality QTLs. The fruit quality gene map developed here is a valuable tool for dissecting the genetic architecture of fruit quality traits in *Prunus *crops.

## Background

Molecular genetic linkage maps have become a major tool in genetics, genomics and breeding of plant and animal species. Linkage maps provide opportunities for unlocking the complex genetics of quantitatively inherited traits through the localization of quantitative trait loci (QTL), identification and positional cloning of individual genes, development of genome-wide physical maps, assembly and annotation of whole genome sequence, and serve as a repository of markers useful in marker-assisted breeding (MAB) of crop and animal species. Among the most informative maps for MAB are those constructed using parent genotypes directly involved in breeding programs.

Peach is one of the best genetically characterized species in the Rosaceae family [1,2], and the most economically important crop in *Prunus *[1], a genus that also includes nectarine, plum, apricot, cherry, and almond. The small genome size and expanding genomic resources of peach highlight peach as a model species for genomics studies of tree fruits [1-4]. Details of these genetic and genomic resources are updated and described on the Genomic Database for Rosaceae (GDR) [5].

While numerous *Prunus *species linkage maps have been published, the interspecific linkage map (T × E) developed from an interspecific cross of almond ("Texas") with peach ("Earlygold") is the most saturated of all these linkage maps [6-8]. Due to this saturation, a high degree of polymorphism, and extensive co-linearity and synteny among *Prunus *genomes [9,10], research community consensus has established the T × E map as the reference map for all *Prunus *species. The most recent published version of the T × E map contains 562 markers spanning 519 cM with an average density of 0.9 cM per marker [8]. Building on the reference status of T × E, a bin-mapping strategy was developed [11]. In this technique, recombination patterns in six progeny of the T × E mapping population were used to reduce the *Prunus *genome to 67 "bins" of 7.8 cM average length and to further populate the reference map with an additional 264 microsatellite-derived markers [11]. Other interspecific *Prunus *linkage maps were derived from almond 'Padre' × peach selection 54P545 [12,13], and myrobalan plum clone P.2175 × almond-peach hybrid clone GN22 [14]. Interspecific maps are easily saturated with markers due to the high level of polymorphism between parent genotypes. However, they are limited in their immediate applicability to cultivar improvement via MAS when compared to intraspecific maps because markers that are polymorphic between species are often not polymorphic within species. This is especially true for peach which has a narrow genetic base [15]. Reported intraspecific *Prunus *linkage maps include those of almond [16-18], apricot [19-22], sweet cherry [23], and peach [15,24-29]. The ultimate stated goal of most linkage map construction efforts for *Prunus *crop species is the development of breeder-friendly MAB tools. Potential benefits of MAB are particularly great for these crop species because of their long juvenility and requirements for large field planting spaces.

The concept of fruit quality of *Prunus *fruit crops includes both its attainment, such changes in color, flavor, and texture as fruit develop, grow, and ripen, and its maintenance following harvest from the tree as the perishable tissues senesce. *Prunus *fruit development, growth, ripening, and senescence includes major biochemical and sensory changes in texture, color, and flavor. The genetic dissection of these complex processes has important applications in crop improvement, to facilitate maximizing and maintaining stone fruit quality from production and processing through to marketing and consumption.

The goal of the present study was to develop a genomic resource to facilitate the genetic dissection of *Prunus *fruit quality traits. This paper reports the genetic mapping in the *Prunus *genome of candidate genes for fruit texture, pigmentation, flavor, and cold-responsiveness of peach, using both an intraspecific peach population to create a linkage map for genetic analyses of fruit quality and chilling injury (CI), and the interspecific *Prunus *reference map. The utility of the "fruit quality gene map" developed here for *Prunus *is demonstrated by highlighting co-localization of fruit quality QTLs with mapped fruit quality candidate genes.

## Results

### Morphological markers

Peach blossom petals can be large and showy or small and curved on margins (non-showy). Non-showy is dominant to showy [30]. 'Dr. Davis' and 'Georgia Belle' are both heterozygous and therefore non-showy for this locus. Pop-DG progeny segregated as 115 non-showy: 37 showy, fitting the expected Mendelian ratio of 3:1 (*χ*^2 ^= 0.04; *P *= 0.98). This trait was designated *Sh *and mapped to the middle of linkage group G8 of Pop-DG and flanked by SSR marker CPPCT006 at 5 cM above and CI resistance CG marker *Unk5 *at 11.2 cM below. Segregation and mapping of the peach mesocarp color (*Y*) and the freestone melting flesh (*F-M*) loci have been reported previously for Pop-DG [31,32].

### Molecular marker polymorphism in Pop-DG

**Figure 1:**
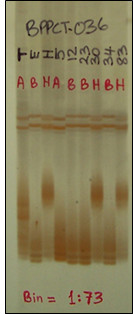
**Polyacrylamide gel profile of SSR marker BPPCT036**. The gel profile shows the re-assignment of BPPCT036 from linkage group G4 (bin 4:63) to bin 1:73 on the *Prunus *T × E reference map.

Three types of reproducible marker polymorphism were observed on the PAGE profiles of the CG PCR products: fragment size polymorphism of the targeted gene fragments, additional markers that were designated as "CG accessory markers" generated elsewhere in the profile than the main CG fragments, and single strand conformation polymorphism (SSCP). Under the conditions used for PAGE, some reproducible sharp or shadowy banding patterns were observed in association with the target PCR product. We have proved this to be mobility shifts characteristic of SSCP resulting from SNPs within the amplicons [33]. The SSCP phenomenon also occurred for many SSRs (Figure 1).

**Figure 2:**
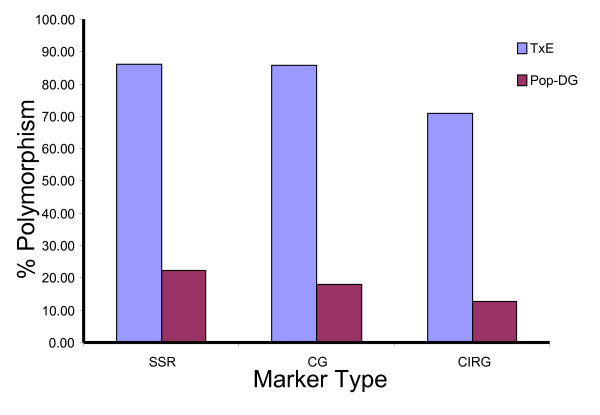
**Level of polymorphism of three molecular marker types in Pop-DG and T × E**. The intraspecific peach population (Pop-DG) compared with the interspecific *Prunus *population (T × E); SSR = simple sequence repeat, CG = fruit ripening candidate genes, CIRG = CI resistance genes.

Of all 229 SSR primer pairs screened for polymorphism in Pop-DG, 68 (~30%) were polymorphic and produced 79 SSR markers (Figure 2). Polymorphism was higher in the 133 genomic SSRs (37%) than in the 96 EST-SSRs (23%). Of the 76 novel ChillPeach EST-SSRs tested in Pop-DG, 17 (22%) were polymorphic, on par with the public EST-SSR polymorphism. The remaining 59 ChillPeach EST-SSRs were screened on the T × E bin set, out of which 51 (86%) were polymorphic. Approximately 13% and 18% polymorphism was obtained for CI resistance CGs and other CGs in Pop-DG, respectively, while 71% and 86% polymorphism was obtained in T × E, respectively (Figure 2). Marker polymorphism in Pop-DG was therefore at least four to five times less than in T × E for all classes of marker tested.

### Pop-DG linkage map

**Figure 3:**
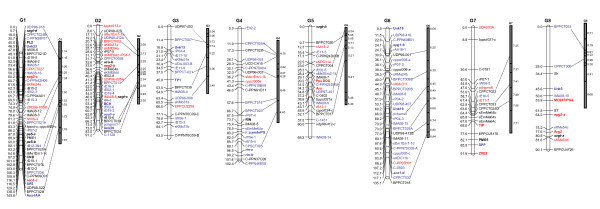
**Genetic linkage map of Pop-DG with fruit texture, flavor, pigment, and CI resistance genes**. Pop-DG = 'Dr. Davis × 'Georgia Belle'. Open vertical bars represent linkage groups. Vertical solid bars represent linkage groups of the T × E *Prunus *reference map (Dirlewanger et al. 2004; Howard et al. 2005) showing the bins and anchored with linkage groups of Pop-DG. Positions of SSR markers on the T × E map corresponding to the Pop-DG map are connected by dotted lines. Genetic markers are to the right side of each linkage group of Pop-DG, genetic distances (cM) are to the left. Markers in bold are fruit texture, pigment, flavor, and CI resistance candidate genes. Markers with prefix 'C-' are novel *Prunus *EST-SSRs obtained from the ChillPeach database (Ogundiwin et al. 2008). RAF and SRAP markers start with prefixes 'r' and 's', respectively. Accessory markers are italicized. Markers in blue fonts were heterozygous in 'Georgia Belle' only, markers in red fonts were heterozygous in 'Dr. Davis' only, and all other markers were heterozygous in both parents.

The Pop-DG intraspecific peach linkage map contained a total of 211 markers (208 molecular and three morphological) distributed over eight linkage groups corresponding to the haploid chromosome number of peach (Figure 3). The map covered 818.2 cM of the peach genome with an average of 4.0 cM interval between markers. The markers on Pop-DG map consisted of three Mendelian trait loci, 24 CGs, 79 SSRs, 40 RAFs, 23 SRAPs, 14 IMAs, and 28 CG accessory markers associated with CGs. Of the 79 SSR markers on Pop-DG, 39 were shared with the published *Prunus *T × E reference map (Figure 3). These common markers enabled the determination of linkage group orientation and assignment of linkage group numbers for the Pop-DG map. Shared markers were co-linear between Pop-DG and T × E except in three cases. Marker positions for BPPCT024, BPPCT030, and pchgms1 were inverted at the lower end of linkage group G2 of Pop-DG compared to G2 of T × E, positions of BPPCT021 and UDP96-008 were inverted in the middle of G3 of Pop-DG compared to G3 of T × E, and positions of BPPCT026 and CPPCT004 were inverted towards the upper end of G5 of Pop-DG compared to G5 of T × E. One SSR marker (BPPCT036) that was originally placed on linkage group G4 of T × E [7] mapped to the distal end of G1 in Pop-DG. To resolve this discrepancy, BPPCT036 was tested on the T × E bin set which confirmed its true location in bin 1:73 (Figure 1), corresponding to its position on the Pop-DG linkage map.

The features of CGs mapped to Pop-DG are presented in Table 1. Eleven markers were derived from fruit quality-related CGs (Table 1), seven of which were texture CGs. One of these texture CGs, *endoPG *(endopolygalacturonase), mapped to linkage group G4 as previously reported (Peace et al. 2005a). The others were two pectin methylesterases (*PME1 *&*PME5*) on G1 and G7, another polygalacturonase (*PG4*) on G8, pectate lyase (*PL2*) on G1, alpha-L-arabinofuranosidase (*Ara*) on G5, and a MADS box transcription factor similar to tomato ripening inhibitor (*RIN*) on G4. Three pigmentation CGs mapped to Pop-DG. These were beta-carotene hydroxylase (*BCH*) on G2, leucoanthocyanidin dioxygenase (*PpLDOX*) on G5 as previously reported by Ogundiwin et al. (2008), and zeaxanthin epoxidase (*ZXE2*) on G7. A flavor CG for sucrose phosphate synthase (*SPS*) mapped to G1. Thirteen mapped Pop-DG gene markers were obtained from CI resistance CGs. Seven of these have functional annotation: Aux/IAA protein (*Aux-IAA*) on G1, chloroplast nucleoid DNA binding (*CND*) on G1, thaumatin-like protein1 precursor (*TP1*) on G3, serine protease-like protein (*SPP*) on G7, tonoplast intrinsic protein (*TIP*) on G7, indole-3-acetic acid-induced protein ARG2 (*Arg2*) on G8, and sulfate transporter (*ST*) on G8. The other six had no functional annotation (labeled with the prefix "Unk" for "unknown") on G1, G2, G3, G8, and two on G6. Some dominant markers were generated from accessory fragments amplified alongside major amplicons of a few candidate genes (See Additional file 1 - Table S1). Seventeen of such markers were mapped to Pop-DG.

**Table 1:** Features of candidate and cold responsive genes mapped to Pop-DG

LG	Marker Code	Functional Annotation	Clone/Accession #	EST Source	CG type^a^
1	PL2	Pectate lyase	BU041363	GDR	Texture
	Unk23	similar to F19P19.4 protein related cluster	PP1004A08-T7_c_s	ChillPeach	CIRG
	PME1	pectinesterase, putative	BU043277	GDR	Texture
	CND	Chloroplast nucleoid DNA binding protein related cluster	PPN018D10-T7_c_s	ChillPeach	CIRG
	SPS	Sucrose phosphate synthase	DY653691	GDR	Flavor
	Aux-IAA	Aux/IAA protein related cluster	CL78Contig1	ChillPeach	CIRG

2	BCH	Beta-carotene-hydroxylase	BU044761	GDR	Pigment
	Unk20	OSJNBb0039L24.13 protein	CL1095Contig1	ChillPeach	CIRG

3	Unk13	highly similar to OSJNBb0004A17.4 protein related cluster	CL32Contig2	ChillPeach	CIRG
	TP1	Thaumatin-like protein 1 precursor	PPN003H07-T7_c_s	ChillPeach	CIRG

4	RIN	Similar to *Solanum lycopersicum *MADS-RIN MADS box transcription factor	BU045116	GDR	Texture
	endoPG	endopolygalacturonase	BU040689	GDR	Texture

5	PpLDOX	Leucoanthocyanidin dioxygenase	EU292217	Ogundiwin et al., 2008	Pigment
	Ara	Alpha-L-arabinofuranosidase	DQ486870	NCBI	Texture

6	Unk10	No annotation available	PP1005B10-T7_c_s	ChillPeach	CIRG
	Unk19	No annotation available	PPN024C05-T7_c_s	ChillPeach	CIRG

7	SPP	Serine protease-like protein related cluster	PPN007C09-T7_c_s	ChillPeach	CIRG
	PME5	pectin methylesterase - like protein	BU044844	GDR	Texture
	TIP	Tonoplast intrinsic protein related cluster	PP1003C07-T7_c_s	ChillPeach	ChillPeach
	ZXE2	Zeaxanthin epoxidase	CL377Contig1	ChillPeach	Pigment

8	Unk5	No annotation available	PP1004F11-T7_c_s	ChillPeach	CIRG
	PG4	*P. persica *PG gene	X77231	NCBI	Texture
	ST	Sulfate transporter 3.1	PPN065F08-T7_c_s	ChillPeach	CIRG
	Arg2	Indole-3-acetic acid-induced protein ARG2 related cluster	CL704Contig1	ChillPeach	CIRG

^a^: CIRG = chilling injury resistance genes

Features of the 18 ChillPeach ESTs that produced 21 EST-SSRs mapped to Pop-DG are provided (See Additional file 2 - Table S2). Eight of these have annotated functions: two zinc finger-RING type, thioredoxin domain 2, POZ/BTB containing protein, biotin synthase, lysine ketoglutarate reductase, transfactor-like protein, TRNA intron endonuclease, and farnesyltransferase beta subunit. Other ChillPeach EST-SSRs on Pop-DG have no known functional annotation.

### Bin-mapping CGs and ChillPeach EST-SSRs to the *Prunus *reference T × E map

**Figure 4:**
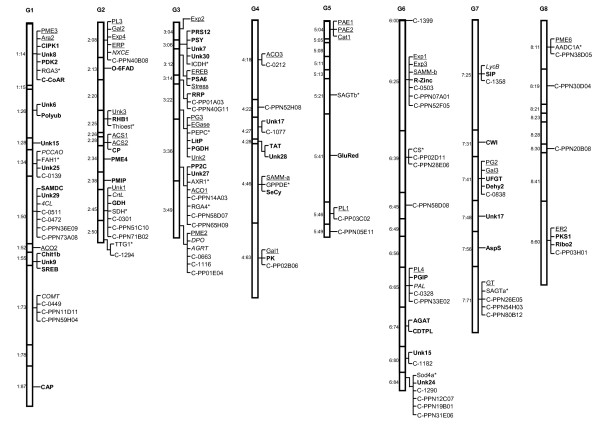
**Candidate genes (CGs) and novel *Prunus *EST-SSR markers bin-mapped to the *Prunus *T × E reference map**. CI resistance CGs are in bold fonts, texture CGs are underlined, CGs related to fruit pigmentation are italicized, other CGs are asterisked, and new *Prunus *EST-SSRs are in normal font.

The bin-mapping technique developed for the *Prunus *T × E reference map enabled the mapping of 158 markers that were monomorphic in Pop-DG (Figure 4; See Additional file 3 - Table S3). These included 109 CGs and 49 novel ChillPeach EST-SSRs. The bin-mapped CGs included 35 texture and nine pigmentation CGs. All markers mapped to 53 of the 67 T × E bins. Approximately 30 of the 50 bin-mapped ChillPeach EST-SSR markers (60%) were derived from ESTs with known GO annotations (See Additional file 2 - Table S2). Others were unknown.

## Discussion

We have developed a detailed fruit quality and ripening gene map for *Prunus*. The fruit quality gene map contains 133 candidate genes (CGs) implicated in fruit ripening, softening, flavor, and pigmentation, and chilling injury resistance. The Pop-DG peach map is almost entirely co-linear with the *Prunus *reference T × E map such that locations of markers and quantitative trait loci (QTLs) located on Pop-DG can be readily cross-referenced to T × E and other *Prunus *maps aligned to T × E. Similarly, markers and QTLs in other *Prunus *maps (and other Rosaceae crop maps as comparative genomics advances in this family) can be compared to the *Prunus *fruit quality gene map to identify genes controlling fruit ripening and sensory quality.

### Co-linearity between Pop-DG and T × E maps

Using a reference genetic map with available bin-mapping resources to map genes and other DNA sequences not polymorphic within crop-specific maps is a powerful means of identifying marker-trait associations. The degree of resolution offered by bin-mapping in T × E (1 to 30 cM bins) is at the same scale as typical QTL mapping, such that detection of co-location (and equally useful, lack of co-location) of candidate genes with previously mapped QTLs is readily achievable. The remarkable conservation of co-linearity among the genomes of *Prunus *species [8] was exploited by bin-mapping to the T × E reference map many CGs that were monomorphic in Pop-DG. The higher rate of polymorphism observed in T × E compared to Pop-DG is not surprising because T × E is an F_2 _population from an interspecific cross. Pop-DG's polymorphism results from a relatively high heterozygosity in 'Georgia Belle' compared to modern cultivars, low heterozygosity in 'Dr. Davis' arising from a pedigree of closely related yellow non-melting canning peaches, and divergent breeding histories of these two parent cultivars. We expect that intraspecific peach populations derived from modern cultivars within the same market type (fresh or canning) will display even less polymorphism than Pop-DG. Inversion of marker positions observed between Pop-DG and T × E in three locations (linkage groups G2, G3, and G5) were among marker pairs that were close together on the T × E map (< 10 cM), suggesting that they are more probably caused by errors in the assignment of marker order than to inversion of chromosome fragments [8].

### Simply inherited traits mapped to Pop-DG

Markers on the Pop-DG linkage map included three simply-inherited Mendelian quality and morphological traits: freestone/clingstone and melting flesh/non-melting flesh (*F-M*), mesocarp color (*Y*), and non-showy flower petals (*Sh*). The *Sh *locus mapped to linkage group G8 of Pop-DG. This is the first report of its genomic location since its inheritance was elucidated in the early to mid 1940s. Confirmation of its location on G8 is emerging from some unpublished results (Shenghua Fan and Tatyana Zhebentyayeva, personal communication). This is the only Mendelian trait mapped to G8 of *Prunus *to date. Linkage group G8 has proved difficult to map in some intraspecific peach mapping efforts [29,34]. In this report also, G8 was the linkage group with fewest number of markers. This could partly explain why it took so long to determine the genomic location of the *Sh *locus. The *F-M *(on linkage group G4) and *Y *(on linkage group G1) loci are among the list of 28 simply-inherited Mendelian traits mapped to *Prunus *genome in prior studies [8], and their locations are confirmed in Pop-DG.

### Novel *Prunus *EST-SSR markers

Seventy new *Prunus *EST-SSR markers were mapped either directly to Pop-DG (21 markers) or bin-mapped to the T × E reference map (49 markers). The markers were obtained from the ChillPeach EST database [35]. The ChillPeach database is a specialized collection of ESTs from peach mesocarp tissue subjected to cold storage and ripening. The new set of mapped EST-SSR markers provides additional resources for molecular marker analyses in *Prunus *species.

### Identity of mapped CGs

CG sequence identities were confirmed for *endoPG *[32], *PpLDOX *[33], and *RIN *(data not shown), where fragment lengths and DNA sequence of amplicons corresponded to original EST sequences. The identity of remaining CGs was confirmed by fragment length, where the most intense amplicons were either the expected size (approximately two-thirds of CGs) or were 80-1300 bp larger presumably due to the inclusion of one or more introns (and confirmed for the aforementioned cases). Identity of CG amplicons was also supported in many cases by previously reported map locations (described below). Further validation via sequencing would be desirable for the remaining CGs, and is the next recommended step for pursuit of specific QTL candidates for fruit quality traits of interest.

### Comparing locations of CGs bin-mapped on T × E to other *Prunus *maps

Several previously mapped fruit candidate genes in *Prunus *corresponded to their genome locations in the present study. The locations of three CGs bin-mapped to T × E in this study corresponded to a previous interspecific 'Padre' × 54P455 map [13]. Catalase was mapped as an isozyme locus (*Cat1*) to the top of linkage group G5 of the 'Padre' × 54P455 map. We bin-mapped this texture CG to T × E bin 5:04. In earlier studies, the peach fruit acidity locus (*D*) was mapped to the top of G5 [25] and a tight linkage between *Cat1 *and the *D *locus was also reported [36]. Another isozyme, isocitrate dehydrogenase, was mapped as *ICD *to the top of linkage group G3 of 'Padre' × 54P455 [13] and as Icdh1 to T × E [37], and we bin-mapped this flavor CG as *ICDH *to T × E bin 3:06. Dehydrin was mapped as *DHN1 *and *Dehy2 *to the middle of group G7 of 'Padre' × 54P455 [13] and T × E (in this report), respectively.

The gene encoding polygalacturonase inhibiting protein (*PGIP*) mapped to different locations in the two studies; it mapped to G7 in 'Padre' × 54P455 and to G6 on T × E. Different members of the *PGIP *gene family may have been mapped in the two separate studies. Another independent study bin-mapped the genes *endoPG *(as EPPCU1775) to bin 4:63, *ACO1 *(as MD206a) to 3:36, *ACO3 *(as MD205a) to 4:18, and *PG4 *(as MD207a) to 8:63 to T × E [11]. *PG4 *was also mapped as isozyme *PG *to the same end of G8 in an almond linkage map [16]. *PEPC *was mapped as *PEPc *in a 'Ferjalou Jalousia' × 'Fantasia' map [37] in the same region as our *PEPC *(3:22). These authors [37] also reported the location of another ten candidate genes for sweetness and acidity, and a different expansin gene to those mapped here, to various locations on a peach map using the T × E population. Three further fruit quality CGs were mapped in a separate study [11]: NADP dependent sorbitol 6-phosphate dehydrogenase (as MD201a) for sweetness to bin 8:19, H+ ATPase (as MD203a) for sweetness/acidity to bin 1:73, and endo-beta-1,4-glucanase for texture to bin 5:41 (a different gene family member to our *EGase *of bin 3:22). Additional fruit quality candidate genes have been located in the *Prunus *genome during hybridization-based efforts to physically map peach ESTs [4].

### Co-locations of candidate genes and chilling injury QTLs

The co-linearity between the Pop-DG and T × E maps has begun to yield benefits in dissecting the genetic control of fruit quality traits in peach. We are using the fruit quality gene map to better understand the genetics of resistance to chilling injury, particularly focusing on the major symptoms of mealiness, browning, and bleeding. At least two cases of co-localizations of chilling injury QTLs and CGs mapped to Pop-DG/T × E were previously reported: *endoPG *on G4 with major QTLs for mealiness and bleeding [31,38,39] as well as to the *Freestone-Melting flesh *locus [31,32] and *PpLDOX *on G5 with cold storage-induced browning [33].

#### Mealiness

A peach homolog of tomato MADS-RIN ripening inhibitor gene (*RIN*), necessary for fruit ripening in tomato, mapped close (6 cM) to *endoPG*. Two *RIN *accessory markers (*rin-a *and *rin-b*) also mapped distally to *endoPG*. The functional role of *RIN *in peach fruit ripening, mealiness, or bleeding has not yet been established. Four additional minor QTLs were reported for mealiness in Pop-DG corresponding approximately to T × E bins 4:18, 4:22-4:27, 4:28, and 6:80-6:84 [39]. We mapped one texture CG (*ACO3*) to bin 4:18, one CI resistance CG (*Unk17*) to bin 4:27, two CI resistance CGs (*TAT *and *Unk28*) to bin 4:28, and two CI resistance CGs (*Unk15 *and *Unk24*) to bins 6:80-6:84. *ACO3 *encodes 1-aminocyclopropane-1-carboxylate oxidase, a critical enzyme in ethylene biosynthesis. *TAT *(tyrosine aminotransferase) is an inducible protein in the plant methyl jasmonate defense system [40]. *Unk15*, *Unk17*, *Unk24*, and *Unk28 *are genes of unknown functions that were differentially regulated in cold-treated peach mesocarp tissue [35]. Each of these CGs are potential markers for mealiness resistance and warrant further investigation.

#### Flesh browning

Some CGs mapped to locations where QTLs have been reported for cold storage-induced flesh browning in peach fruit. A major browning QTL was located on linkage group G5 of Pop-DG corresponding to bin 5:21 of T × E [33,39]. *PpLDOX *(leucoanthocyanidin dioxygenase), which was initially mapped to this bin, was later fine-mapped to Pop-DG, and evidence of its association with browning was established [33]. The two minor browning QTLs reported on linkage group G2 of Pop-DG [39] correspond roughly to bins 2:08-2:13 and 2:20-2:25 of T × E map. Four ripening CGs (*PL3*, *Gal2*, *Exp4*, and *ERP*) and one pigmentation CG (*NXCE*) mapped to bin 2:08, and one CI resistance CG (*O-6FAD*) mapped to bin 2:13. Also one ripening CG (*Unk3*) and one CI resistance CG (*RHB1*) mapped to bin 2:25. *PL3 *(pectate lyase), *Gal2 *(beta-galactosidase), and *EXP4 *(expansin) are cell wall-degrading enzymes while *ERP *(ethylene-responsive small gtp-binding) is an ethylene-related protein. *Unk3 *is a protein of unknown function which was up-regulated by ethylene in peach mesocarp tissue [41]. *NXCE *(neoxanthin cleavage enzyme) is an abscisic acid biosynthesis gene that acts by oxidative cleavage of a carotenoid neoxanthin [42]. *O-6FAD *(omega-6 fatty acid desaturase) and *RHB1 *(RING-H2 finger protein) were up-regulated in cold-treated peach mesocarp tissue [35]. These CGs may be useful markers for developing resistance to cold-induced browning in stone fruit.

#### Cold-induced bleeding

In addition to the major QTL detected in peach fruit at the *F-M*/*endoPG *locus on G4 of Pop-DG, two minor QTLs were also reported for cold-induced bleeding on Pop-DG linkage group G1 [39], corresponding to T × E bins 1:14 and 1:34. A texture CG (*PL2*) and a CI resistance CG (*Unk23*) mapped close to the minor bleeding QTL peak at the top end of G1. Two texture CGs (*PME3 *and *Ara2*) and four CI resistance CGs (*CIPK1*, *PDK2*, *C-CoAR*, and *Unk8*) mapped to bin 1:14, while *PCCAO *(pigmentation CG) and *Unk25 *(CI resistance CG) mapped to bin 1:34. *PL2 *(pectate lyase), *PME3 *(pectin methylesterase), and *Ara2 *(alpha-L-arabinofuranosidase) are cell wall-degrading enzymes [41,43]. *PPCAO *(peroxisomal copper-containing amine oxidase) catalyzes the oxidation of amines to aldehyde, ammonia and hydrogen peroxide [44]. *CIPK1 *(calcineurin B-like protein-interacting protein kinase), involved in plant calcium signaling [45], *PDK2 *(pyruvate dehydogenase kinase), *C-CoAR *(cinnamoyl-CoA reductase), *Unk8*, and *Unk25 *were up-regulated in cold-treated mesocarp tissue of peach [35]. These CGs will be further investigated for possible roles in the formation of bleeding and developing bleeding-free peach cultivars.

### Inferred co-locations of candidate genes and other published fruit quality traits

Several CGs mapped to genomic regions corresponding to fruit quality QTLs reported in other studies. Examples are those reported by [34] and [46].

#### Putative candidate genes for fruit quality QTLs on P1908 × 'Summergrand' map [34]

On linkage group G1 QTLs were detected by [34] for fructose (*Fru*_1,2_), sweetness (*Swe*_2_), quinic acid (*Qui*_1_), fruit cheek diameter (*FCheekD*_2_), fruit mass (*FMass*_2_), and fruit suture diameter (*FSutureD*_2_) in the region corresponding to the T × E bin 1:26 on which we bin-mapped *Polyub *(Polyubiquitin) and *Unk6*; QTLs for citric acid (*Cit*_1_) and total sugar (*TSugar*_1_) in the region equivalent to bin 1:50 of T × E on which we bin-mapped SAMDC (S-adenosylmethionine decarboxylase), *Unk29*, *4CL *(4-coumarate-CoA ligase-like protein), *C-0472 *(6-phosphogluconolactonase), *C-PPN36E09*, *C-PPN73A08*; QTLs for total sugar (*TSugar*_2_) and quinic acid (*Qui*_2_) in the region corresponding to bin 1:52-1:55 of T × E where we bin-mapped *Chitb *(Chitinase Ib), *Unk9 *and *SREB *(Sucrose-responsive element binding protein). Notable among these gene markers are those encoding sucrose-responsive element binding protein (*SREB*) and phosphogluconolactonase (*C-0472*) for their possible involvement in sugar biosynthesis [47] and inducible expression [48], respectively. In addition, [34] localized QTLs for sucrose (*Suc*_1_, *Suc*_2_), glucose (*Glu*_2_), and quinic acid (*Qui*_1_) on linkage group G7 region comparable to bin 7:56 of T × E containing bin-mapped CG *AspS *(Asparagine synthetase). The expression of *AspS *has been shown to increase with a decrease in sucrose levels [49].

The following QTLs were also detected for peach fruit quality on linkage group G4 by [34]: fruit mass (*FMass*_2_), fruit polar diameter (*FPolarD*_2_), soluble solid concentrate (*SSC*_1,2_) and juiciness (*Jui*_2_) on a region corresponding to bin 4:18 of T × E on which *ACO3 *and *C-0212 *(Acetyl Co-A acetyltransferase) were bin-mapped; and citric acid (*Cit*_2_), quinic acid (*Qui*_2_), total acid (*TAcid*_2_), sorbitol (*Sor*_1,2_), fructose (*Fru*_1_) and malic acid (*Mal*_1_) on a region equivalent to bin 4:27 - 4:28 of T × E on which were bin-mapped *Unk17*, *Unk28*, *C-1077*, and *TAT *(putative tyrosine aminotransferase). The authors also localized the following peach fruit quality QTLs on linkage group G5: glucose (*Glu*_2_), fruit suture diameter (*FSutureD*_2_), and fruit cheek diameter (*FCheekD*_2_), as well as the major locus controlling fruit acidity, *D*, on a region corresponding approximately to bin 5:04 of T × E on which we bin-mapped *PAE1*, *PAE2*, and *Cat1*; red skin coloration (*SRColor*_2_), dry flesh mass content (*DFMC*_1_), soluble solid concentrate (*SSC*_1_), fruit mass (*FMass*_1_), fruit polar diameter (*FPolarD*_1,2_), and fruit suture diameter (*FSutureD*_1_) on bin 5:46-5:49 of T × E on which *PL, C-PP03C02*, and *C-PPN05E11 *(SufE-like protein) were bin-mapped. SulfE-related proteins have been implicated in Fe-S metabolism and export [50].

#### Putative candidate genes for fruit quality QTLs on 'Ferjalou Jalousia' × 'Fantasia' map [46]

A number of fruit quality QTLs (including fresh weight, sucrose, and SSC) were detected by [46] on linkage group G6 of 'Ferjalou Jalousia' × 'Fantasia' map, on the region corresponding to bin 6:74-6:84 of the T × E linkage map. To this bin, we mapped *AGAT *(Alanine--glyoxylate aminotransferase), *CDTPL *(C-terminal domain phosphatase-like), *Sod4a *(Superoxide dismutase), *Unk5*, *Unk24*, *C-1182 *(BZIP transcription factor bZIP105), *C-1290*, *C-PPN12C07*, *C-PPN19B01*, and *C-PPN31E06 *(Glutamine-fructose-6-phosphate transaminase). Glutamine-fructose-6-phosphate transaminase is an important enzyme in biosynthesis of amino sugar-containing macromolecules [51]. Also on linkage group G4, Dirlewanger et al (1999) localized QTLs for SSC and fructose to a region equivalent to the T × E bin 4:46. The candidate genes mapped to this bin were *SeCy *(Sesquiterpene cyclase), *SAMM *(S-adenosylmethionine:2-demethylmenaquinone methyltransferase), and *GPPDE *(glycerophosphoryl diester phosphodiesterase).

## Conclusion

We have developed a fruit quality gene map for *Prunus *by determining the genomic locations of 133 fruit quality candidate genes with an intraspecific peach population, Pop-DG, and the *Prunus *reference map, T × E. Sufficient SSR marker anchoring between both maps allowed easy cross-referencing of marker and trait locus positions. We demonstrate here the use of this gene map for dissecting the molecular genetics of CI in peach. Using the results of microarray experiments that studied gene expression in cold-treated peach mesocarp tissue, 63 cold-responsive genes were located on the fruit quality gene map, allowing the detection of co-locations of these CI resistance CGs with QTLs for CI symptoms. We also highlight new CGs for previously reported *Prunus *QTLs of other fruit quality traits. The fruit quality gene map presented here is expected to be a valuable resource for the genetic analysis of fruit ripening and related fruit quality traits in *Prunus *species.

## Methods

### Mapping population and T × E bin set

Pop-DG is a peach intraspecific cross between 'Dr. Davis' (female parent) and 'Georgia Belle' (pollen parent). 'Dr. Davis' is a modern canning peach cultivar while 'Georgia Belle' is a century-old fresh market peach cultivar. These cultivars contrast for many fruit quality and other traits (Table 2, 3). Pop-DG, created and managed to study the genetics of fruit quality attainment and maintenance, particularly resistance to CI, in peach and nectarine, was established in two nearby orchards at Kearney Agricultural Center (Parlier, CA, USA). The first orchard was established in 1998 containing 51 verified hybrids [32]. Each progeny genotype was represented by two trees in the orchard; one tree planted on its own roots and the other tree on 'Nemaguard' rootstock. The second orchard was established in 2002, containing single trees of 277 true hybrid progeny on their own roots. All 51 progeny of the first orchard and 101 progeny of the second orchard (152 true hybrid progeny total) were used for Pop-DG linkage mapping. For bin-mapping in T × E, DNA samples of the bin set of the 'Texas' × 'Earlygold' population ('Earlygold' the F_1 _plant, and six F_2 _plants) [11], kindly provided by Dr. Werner Howad, were used. We also included the second parent - 'Texas' in each test.

**Table 2:** Fruit quality attributes of Pop-DG parent cultivars, 'Dr. Davis' and 'Georgia Belle'

Trait^a^	'Dr. Davis'	'Georgia Belle'
Ripening date	Later	Earlier
Skin color	Blush on orange ground	Green/yellow, no blush
Flesh color	Yellow-orange	White-cream
Stone adhesion	Clingstone	Freestone
Flesh texture	Firm, non-melting flesh	Soft, melting flesh
Aroma	Bland	Sharp
Sweetness (SSC)	11.5	13.0
Acidity (TA)	High	Low
Mealiness	None	High susceptibility
Browning	Medium susceptibility	High susceptibility
Bleeding	High susceptibility	Low susceptibility

^a^: TA = titratable acidity, SSC = soluble solids concentrate

### Morphological markers

Pop-DG segregated for three Mendelian morphological traits: freestone melting flesh/clingstone non-melting flesh, white/yellow flesh color, and non-showy/showy flower petals. These traits were scored visually and included in linkage analysis alongside molecular markers.

### Molecular markers

Various classes of molecular markers were evaluated. These marker classes consisted of genomic sequence-derived simple sequence repeats (SSR), expressed sequence tag [EST]-derived SSRs (EST-SSR), ethylene-related candidate genes (CG), texture CGs, pigmentation CGs, flavor CGs, CI resistance CGs, sequence-related amplified polymorphisms (SRAP), randomly amplified DNA fingerprinting (RAF), and inter-microsatellite amplification (IMA).

Candidate genes were nominated from published works and review articles on physiology and biochemistry of fruit ripening, softening, color (pigmentation), and flavor [37,41,43,52-71]. EST sequences of most of the CGs (including those described below) were obtained from the GDR database [5]. Others were obtained from the ChillPeach database [35] and GenBank.

CGs assembled for texture included those putatively encoding ethylene-related enzymes (e.g. aminocyclopropane-1-carboxylate synthases, aminocyclopropane-1-carboxylate oxidases, ethylene receptors, ethylene responsive element binding proteins, s-adenosyl-1-methionine synthases, peptide methionine sulfoxide redutase, and ripening inhibitor protein), although ethylene-related genes are also relevant for other fruit ripening processes. Other texture CGs were those putatively encoding cell wall-degrading enzymes (e.g. polygalacturonases, pectinesterases, pectate lyases, glucanases, mannosidases, xyloglucans, glycosylases and expansins). As indicated by [41], cell wall synthesis enzymes were also included (e.g glycosyltransferases and fiber protein enzymes), and from the same study, several genes of unknown function but strongly up-regulated by ethylene in ripening peach fruit were also included.

For pigmentation (skin and flesh color, including browning and bruising), candidate genes were chosen from the carotenoid and anthocyanin biosynthesis pathways (e.g. neoxanthin cleavage enzyme, leucoanthocyanidin dioxygenase, anthocyanidin-3-glucoside rhamnosyltransferase, beta-carotene hydroxylase, lycopene beta cyclase, peroxisomal copper containing amine oxidase, zeanthin epoxidase, geranylgeranyl pyrophosphate synthase, zeta carotene desaturase and phytoene desaturase). Genes encoding diphenol oxidases and polyphenol oxidases were also included.

Flavor CGs included sugar and acid biosynthesis pathway genes (e.g. sucrose synthase, hydroxyl methylglutaryl CoA reductase, cell wall invertase, sorbitol dehydrogenase, phosphoenolpyruvate carboxylase, chalcone synthase, polyketide synthase, alcohol dehydrogenase, and aromatic amino acid decarboxylase).

CI resistance CGs were cold-responsive genes obtained from the results of microarray analysis of cold-treated peach mesocarp tissues versus untreated mesocarp tissues [35]. Selection of CI resistance CGs were made as follows: 25 top performing genes, 24 genes common to peach and *Arabidopsis thaliana *(ChillPeach microarray data compared with ColdArrayDB: http://cold.stanford.edu/cgi-bin/data.cgi), and 39 genes unique to peach (i.e. not found in ColdArrayDB).

Published reports were the source of all genomic sequence SSRs (see below) and some EST-SSRs, while most EST-SSRs were newly obtained from the ChillPeach EST database [35]. A total of 153 published *Prunus *SSRs were screened for polymorphism between the Pop-DG mapping parents. These were *P. persica *SSRs with the prefix BPPCT [72], CPPCT [73], EPPCU (GDR database: [5]), Pchcms & Pchgms [74], PS [75], UCD-CH [76], and UDP [77,78], and *P. dulcis *SSRs with the prefix UDA [79]. The ChillPeach database provided 76 new EST-SSRs for screening, and the markers were labeled with the prefix 'C-' followed by the clone or contig number (e.g. C-PPN28F07 and C-1128).

RAF markers were obtained according to [80] protocols using Operon decamer primers AA18, B12, B15, D19, E02, E11, E16, P04, P07 and W06 after preliminary screening of many others via the bulked segregant analysis approach [81] with mealiness phenotypic extremes. SRAP marker analysis was conducted according to [82]. One IMA primer (IMA08: (GA)^8^GT; [25]) was also used to generate additional molecular markers for Pop-DG.

### PCR and PAGE

All PCR primers were designed using Primer3 software [83]. EST sequences of CGs were examined for microsatellite motifs, and whenever ESTs were part of a contig, the contig was examined for SNPs and indels. Primers were designed to exploit these polymorphic features. Where these features were not observable, sequences at the 3'-end of the ESTs were used for designing primers to avoid long introns and target less-conserved 3' UTRs. Generally, primers were designed to limit expected amplicon size to ≤ 300 bp such that a PCR product even with an intron as long as 1 kb would still be observable on the large (50 × 38 cm) PAGE plate and 1 bp indels of intron-less amplified fragments could be detected. PCR and PAGE conditions were as reported in Peace et al. (2005b). Primer sequences, annealing temperatures, expected and observed amplicon sizes, and type of polymorphisms are provided (See Additional files 3 and 4).

### Map construction

Linkage analysis was conducted with JoinMap^® ^4 [84]. Linkage parameters were set as 3.0 minimum LOD and 0.45 maximum recombination fraction. The Kosambi mapping function [85] was used to convert recombination fraction to map distances in centimorgans (cM). The marker data type was set as cross-pollination (CP). The bin-mapping procedure followed [11].

## Authors' contributions

EAO, CPP, TMG, FAB and CHC conceived the study. TMG, FAB and CHC generated and established the Pop-DG mapping population. EAO and CPP conducted molecular marker discovery and genotyping, and EAO carried out linkage analysis, map construction and manuscript preparation. DEP also assisted in data analysis. DEP, CPP and FAB provided a very thorough review of the manuscript. All authors read and approved the final manuscript.

## Supplementary Material

Additional file 1 **Table S1- Features of dominant amplicons (accessory markers) generated alongside the target PCR products of candidate and cold responsive genes mapped to Pop-DG**. The data provided represent information on accession number, map location, and fragment size information of dominant amplicons (accessory markers) generated alongside the target PCR products of candidate and cold responsive genes mapped to Pop-DG. Click here for file

Additional file 2 **Table S2 - Characteristics of 71 new *Prunus *ChillPeach EST-SSR markers mapped to the peach/*Prunus *genome**. The data provided represent information on unigene, map location, and functional annotation of 71 new *Prunus *ChillPeach EST-SSR markers mapped to the peach/*Prunus *genome. Click here for file

Additional file 3 **Table S3 - Characteristics of candidate genes (CGs) bin-mapped to the T × E reference *Prunus *map**. The data provided represent information on the genome location (bin name), marker code, clone/accession number, source of ESTs, and CG class of all CGs bin-mapped to the T × E reference *Prunus *map. Click here for file

Additional file 4 **Table S4 - Primer details for candidate genes and EST-SSRs**. The data provided represent information on the primer sequence, annealing temperature, amplicons size, and type of polymorphisms for candidate genes and EST-SSRs. Click here for file

## References

[B1] Abbott A Georgi L Yvergniaux D Wang Y Blenda A Reighard G Inigo M Sosinski B Peach: The model genome for Rosaceae Acta Hort 2002 575 145 155

[B2] Shulaev V Korban SS Sosinski B Abbott AG Aldwinckle HS Folta KM Iezzoni A Main D Arús P Dandekar AM Lewers K Brown SK Davis TM Gardiner SE Potter D Veilleux RE Multiple models for Rosaceae genomics Plant Physiol 2008 157 985 1003 10.1104/pp.107.115618 PMC2442536 18487361

[B3] Byrne DH Isozyme variability in four diploid stone fruits compared with other woody perennial plants J Hered 1990 81 68 71

[B4] Zhebentyayeva TN Swire-Clark G Georgi LL Garay L Jung S Forrest S Blenda AV Blackmon B Mook J Horn R Howad W Arús P Main D Tomkins JP Sosinski B Baird WV Reighard GL Abbott AG A framework physical map for the peach, a model Rosaceae species Tree Genet Genomes 2008 4 745 756 10.1007/s11295-008-0147-z

[B5] Jung S Staton M Lee T Blenda A Svancara R Abbott A Main D GDR (Genome Database for Rosaceae): integrated web-database for Rosaceae genomics and genetics data Nucleic Acids Res 2008 36 D1034 D1040 10.1093/nar/gkm803 17932055 PMC2238863

[B6] Joobeur T Viruel MA de Vicente MC Jauregui B Ballester J Dettori MT Verde I Truco MJ Messeguer R Battle I Quarta R Dirlewanger E Arús P Construction of a saturated linkage map for *Prunus *using an almond × peach F2 progeny Theor Appl Genet 1998 97 1034 1041 10.1007/s001220050988

[B7] Aranzana MJ Pineda A Cosson P Dirlewanger E Ascasibar J Cipriani G Ryder CD Testolini R Abbott A King GJ Iezzoni AF Arús P A set of simple-sequence repeat (SSR) markers covering the *Prunus *genome Theor Appl Genet 2003 106 819 825 12647055 10.1007/s00122-002-1094-y

[B8] Dirlewanger E Graziano E Joobeur T Garriga-Caldere F Cosson P Howard W Arús P Comparative mapping and marker assisted selection in Rosaceae fruit crops Proc Natl Acad Sci USA 2004 101 9891 9896 10.1073/pnas.0307937101 15159547 PMC470769

[B9] Arús P Yamamoto T Dirlewanger E Abbott AG Janick J Synteny in the Rosaceae Plant Breeding Reviews 2006 27 Hoboken: Wiley 172 211

[B10] Abbott AG Arús P Scorza R Kole C Peach Genome mapping and molecular breeding in plants 2006 Berlin: Springer 137 156

[B11] Howad W Yamamoto T Dirlewanger E Testolin R Cosson P Cipriani G Monforte AJ Georgi L Abbott AG Mapping with a few plants: using selective mapping for microsatellite saturation of the *Prunus *reference map Genetics 2005 171 1305 1309 10.1534/genetics.105.043661 16118196 PMC1456826

[B12] Foolad MR Arulsekar S Becerra V Bliss FA A genetic map of *Prunus *based on an interspecific cross between peach and almond Theor Appl Genet 1995 91 262 269 10.1007/BF00220887 24169773

[B13] Bliss FA Arulsekar S Foolad MR Becerra V Gillen AM Warburton ML Dandekar AM Kocsisne GM Mydin KK An expanded genetic linkage map of *Prunus *based on an interspecific cross between almond and peach Genome 2002 45 520 529 10.1139/g02-011 12033621

[B14] Dirlewanger E Cosson P Howad W Capdeville G Bosselut N Claverie M Voisin R Poizat C Lafargue B Baron O Laigret F Kleinhentz M Arús P Esmenjaud D Microsatellite genetic linkage maps of myrobalan plum and an almond-peach hybrid - location of root-knot nematode resistance genes Theor Appl Genet 2004 109 827 838 10.1007/s00122-004-1694-9 15241595

[B15] Rajapakse S Belthoff LE He G Estager AE Scorza R Verde I Ballard RE Baird WV Callahan A Monet R Abbott AG Genetic linkage mapping in peach using morphological, RFLP and RAPD markers Theor Appl Genet 1995 90 503 510 10.1007/BF00221996 24173944

[B16] Viruel MA Messeguer R de Vicente MC Garcia-Mas J Puigdomenech P Vargas FJ Arús P A linkage map with RFLP and isozyme markers for almond Theor Appl Genet 1995 91 964 971 10.1007/BF00223907 24169984

[B17] Joobeur T Periam N de Vicente MC King GJ Arús P Development of a second generation linkage map for almond using RAPD and SSR markers Genome 2000 43 649 688 10.1139/gen-43-4-649 10984177

[B18] Sánchez-Pérez R Howad W Dicenta F Arús P Martínez-Gómez P Mapping major genes and quantitative trait loci controlling agronomic traits in almond Plant Breed 2007 126 310 319 10.1111/j.1439-0523.2007.01329.x

[B19] Hurtado MA Vilanova S Romero C Abbott AG Llacer G Badenes ML Genetic linkage maps of two apricot cultivars (*Prunus armeniaca *L.) based on molecular markers Theor Appl Genet 2002 105 182 191 10.1007/s00122-002-0936-y 12582518

[B20] Vilanova S Romero C Abbott AG Llacer G Badenes ML An apricot (*Prunus armeniaca *L.) F_2 _progeny linkage map based on SSR and AFLP markers, mapping plum pox virus resistance and self-incompatibility traits Theor Appl Genet 2003 107 239 247 10.1007/s00122-003-1243-y 12845439

[B21] Lambert P Hagen LS Arús P Audergon JM Genetic linkage maps of two apricot cultivars (*Prunus armeniaca *L.) compared with the almond 'Texas' × peach 'Earlygold' reference map for *Prunus* Theor Appl Genet 2004 108 1120 1130 10.1007/s00122-003-1526-3 15067399

[B22] Dondini L Lain O Geuna F Banfi R Gaiotti F Tartarini S Bassi D Testolin R Development of a new SSR-based linkage map in apricot and analysis of synteny with existing *Prunus *maps Tree Genet Genomes 2007 3 239 249 10.1007/s11295-006-0059-8

[B23] Olmstead JW Sebolt AM Cabrera A Sooriyapathirana SH Iriarte G Wang D Chen CY Knaap E van der Iezzoni AF Construction of an intra-specific sweet cherry (*Prunus avium *L.) genetic linkage map and synteny analysis with the Prunus reference map Tree Genet Genomes 2008 4 897 910 10.1007/s11295-008-0161-1

[B24] Chaparro JX Werner DJ O'Malley D Sederoff RR Targeted mapping and linkage analysis of morphological, isozyme, and RAPD markers in peach Theor Appl Genet 1994 87 805 815 10.1007/BF00221132 24190466

[B25] Dirlewanger E Pronier V Parvery C Rothan C Guye A Monet R A genetic linkage map of peach (*Prunus persica *L. Batsch) using morphological, RFLP, isoenzyme, RAPD, and AFLP markers Theor Appl Genet 1998 97 888 895 10.1007/s001220050969

[B26] Abbott AG Rajapakse S Sosinski B Lu ZX Sossey-Alaoui K Gannavarapu M Reighard G Ballard RE Baird WV Scorza R Callahan A Construction of saturated linkage maps of peach crosses segregating for characters controlling fruit quality, tree architecture and pest resistance Acta Hort 1998 465 41 49

[B27] Lu Z-X Sosinski B Reighard GL Baird WV Abbott AG Construction of a genetic linkage map and identification of AFLP markers or resistance to root-knot nematodes in peach rootstocks Genome 1998 41 199 207 10.1139/gen-41-2-199

[B28] Yamamoto T Shimada T Imai T Yaegaki H Haji T Matsuta N Yamaguchi M Hayashi T Characterization of morphological traits based on a genetic linkage map in peach Breed Sci 2001 51 271 278 10.1270/jsbbs.51.271

[B29] Dirlewanger E Cosson P Boudehri K Renaud C Capdeville G Tauzin Y Laigret F Moing A Development of a second generation genetic linkage map for peach [*Prunus persica *(L.) Batsch] and characterization of morphological traits affecting flower and fruit Tree Genet Genomes 2006 3 1 13 10.1007/s11295-006-0053-1

[B30] Bailey JS French AP The inheritance of blossom type and blossom size in peach Proc Am Soc Hort Sci 1942 40 248 250

[B31] Peace CP Ahmad R Gradziel TM Dandekar AM Crisosto CH The use of molecular genetics to improve peach and nectarine post-storage quality Acta Hort 2005 682 403 409

[B32] Peace CP Crisosto CH Gradziel TM Endopolygalacturonase: a candidate gene for *Freestone *and *Melting flesh *in peach Mol Breed 2005 16 21 31 10.1007/s11032-005-0828-3

[B33] Ogundiwin EA Peace CP Nicolet CM Rashbrook VK Gradziel TM Bliss FA Parfitt D Crisosto CH Leucoanthocyanidin dioxygenase gene (PpLDOX): a potential functional marker for cold storage browning in peach Tree Genet Genomes 2008 4 543 554 10.1007/s11295-007-0130-0

[B34] Quilot B Wu BH Kervella J Genard M Foulongne M Moreau K QTL analysis of quality traits in an advanced backcross between *Prunus persica *cultivars and the wild related species *P*. *davidiana* Theor Appl Genet 2004 109 884 897 10.1007/s00122-004-1703-z 15168024

[B35] Ogundiwin EA Martí C Forment J Pons C Granell A Gradziel TM Peace CP Crisosto CH Development of ChillPeach genomic tools and identification of cold-responsive genes in peach fruit Plant Mol Biol 2008 68 379 397 10.1007/s11103-008-9378-5 18661259

[B36] Monet R Guye A Roy M Dachary N Peach Mendelian genetics a short review and new results Agronomie 1996 16 321 329 10.1051/agro:19960505

[B37] Etienne C Moing A Dirlewanger E Raymond P Monet R Rothan C Isolation and characterization of six peach cDNAs encoding key proteins in organic acid metabolism and solute accumulation: involvement in regulating peach fruit acidity Physiol Plantarum 2002 114 259 270 10.1034/j.1399-3054.2002.1140212.x 11903973

[B38] Peace CP Crisosto CH Garner DT Dandekar AM Gradziel TM Bliss FA Genetic control of internal breakdown in peach Acta Hort 2006 713 489 496

[B39] Ogundiwin EA Peace CP Gradziel TM Dandekar AM Bliss FA Crisosto CH Molecular genetic dissection of chilling injury in peach fruit Acta Hort 2007 738 633 638

[B40] Lopukhina A Dettenberg M Weiler EW Hollander-Czytko H Cloning and characterization of a coronatine-regulated tyrosine aminotransferase from *Arabidopsis* Plant Physiol 2001 126 1678 1687 10.1104/pp.126.4.1678 11500565 PMC117166

[B41] Trainotti L Zanin D Casadoro G A cell wall-oriented genomic approach reveals a new and unexpected complexity of the softening in peaches J Exp Bot 2003 54 1821 1832 10.1093/jxb/erg198 12815031

[B42] Bouvier F D'Harlingue A Backhaus RA Kumagai MH Camara B Identification of neoxanthin synthase as a carotenoid cyclase paralog Eur J Biochem 2001 267 6346 6352 10.1046/j.1432-1327.2000.01722.x 11029576

[B43] Marin-Rodriguez MC Orchard J Seymour GB Pectate lyases, cell wall degradation and fruit softening J Exp Bot 2002 53 2115 2119 10.1093/jxb/erf089 12324535

[B44] Tipping AJ McPherson MJ Cloning and molecular analysis of the pea seedling copper amine oxidase J Biol Chem 1995 270 16939 16946 10.1074/jbc.270.28.16939 7622512

[B45] Luan S The CBL CIPK network in plant calcium signaling Trends Plant Sci 2008 14 37 42 10.1016/j.tplants.2008.10.005 19054707

[B46] Dirlewanger E Moing A Rothan C Svanella L Pronier V Guye A Plomion C Monet R Mapping QTLs controlling fruit quality in peach (*Prunus persica *(L.) Batsch) Theor Appl Genet 1999 98 18 31 10.1007/s001220051035

[B47] Miclet E Stoven V Michels PAM Opperdoes FR Lallemand J-V Duffieux F NMR spectroscopic analysis of the first two steps of the pentose-phosphate pathway elucidates the role of 6-phosphogluconolactonase J Biol Chem 2001 276 34840 34846 10.1074/jbc.M105174200 11457850

[B48] Ishiguro S Nakamura K Characterization of a cDNA encoding a novel DNA-binding protein, SPF1, that recognizes SP8 sequences in the 5' upstream regions of genes coding for sporamin and betaamylase from sweet potato Mol Gen Genet 1994 244 563 571 10.1007/BF00282746 7969025

[B49] Downs CG Somerfield SD Asparagine synthetase gene expression increases as sucrose declines in broccoli after harvest NZ J Crop Hort Sci 1997 25 191 195 full_text

[B50] Goldsmith-Fischman S Kuzin A Edstrom WC Benach J Shastry R Xiao R Acton TB Honig B Montelione GT Hunt JF The SufE sulfur-acceptor protein contains a conserved core structure that mediates interdomain interactions in a variety of redox protein complexes J Mol Biol 2004 344 549 565 10.1016/j.jmb.2004.08.074 15522304

[B51] Milewski S Glucosamine-6-phosphate synthase - the multi-facets enzyme Biochim Biophys Acta Prot Struct Mol Enzymol 2002 1597 173 192 10.1016/S0167-4838(02)00318-7 12044898

[B52] Kader AA Chordas A Evaluating the browning potential of peaches Calif Agr 1984 38 14 15

[B53] O'Neill SD Tong Y Sporlein B Forkmann G Yoder JI Molecular genetic analysis of chalcone synthase in *Lycopersicon esculentum *and an anthocyanin-deficient mutant Mol Gen Genet 1990 224 279 288 10.1007/BF00271562 1980524

[B54] Bonghi C Rascio N Ramina A Casadoro G Cellulase and polygalacturonase involvement in the abscission of leaf and fruit explants of peach Plant Mol Biol 1992 20 839 848 10.1007/BF00027155 1281437

[B55] Martin T Frommer WB Salanoubat M Willmitzer L Expression of an Arabidopsis sucrose synthase gene indicates a role in metabolization of sucrose both during phloem loading and in sink organs Plant J 1993 4 367 377 10.1046/j.1365-313X.1993.04020367.x 8220487

[B56] Cunningham FX Sun Z Chamovitz D Hirschberg J Grantt E Functional analysis of the beta and epsilon lycopene cyclase enzymes of Arabidopsis reveals a mechanism for control of cyclic carotenoid formation Plant Cell 1996 8 1613 1626 10.1105/tpc.8.9.1613 8837512 PMC161302

[B57] Kalaitzis P Solomos T Tucket ML Three different polygalacturonases are expressed in tomato leaf and flower abscission, each with a different temporal expression pattern Plant Physiol 1997 113 1303 1308 10.1104/pp.113.4.1303 9112778 PMC158253

[B58] Hong SB Tucker ML Genomic organization of six tomato polygalacturonases and 5' upstream sequence identity with *tap1 *and *win2 *genes Mol Gen Genet 1998 258 479 487 10.1007/s004380050758 9669329

[B59] Chang S Tan C Frankel EN Barrett DM Low-density lipoprotein antioxidant activity of phenolic compounds and polyphenol oxidase activity in selected clingstone peach cultivars J Agric Food Chem 2000 48 147 151 10.1021/jf9904564 10691607

[B60] Ruperti B Bonghi C Rasori A Ramina A Tonutti P Characterization and expression of two members of the peach 1-aminocyclopropane-1-carboxylate oxidase gene family Physiol Plantarum 2001 111 336 344 10.1034/j.1399-3054.2001.1110311.x 11240918

[B61] Yamada K Niwa N Shiratake K Yamaki S cDNA cloning of NAD-dependent sorbitol dehydrogenase from peach fruit and its expression during fruit development J Hort Sci Biotechnol 2001 76 581 587

[B62] Jaakola L Maatta K Pirttila AN Torronen R Karenlampi S Hohtola A Expression of genes involved in anthocyanin biosynthesis in relation to anthocyanin, proanthocyanindin, and flavonol levels during bilberry fruit development Plant Physiol 2002 130 729 739 10.1104/pp.006957 12376640 PMC166602

[B63] Fridman E Zamir D Functional divergence of a syntenic invertase gene family in tomato, potato, and *Arabidopsis* Plant Physiol 2003 131 603 609 10.1104/pp.014431 12586884 PMC166836

[B64] Iyidogan NF Bayindirli A Effect of l-cysteine, kojic acid and 4-hexylresorcinol combination on inhibition of enzymatic browning in Amasya apple juice J Food Eng 2003 62 299 304 10.1016/S0260-8774(03)00243-7

[B65] Li Y Jones L McQueen-Mason S Expansins and cell growth Curr Opin Plant Biol 2003 6 603 610 10.1016/j.pbi.2003.09.003 14611960

[B66] Trainotti L Pavanello A Zanin D PpEG4 is a peach endo-beta-1,4-glucanase gene whose expression in climacteric peaches does not follow a climacteric pattern J Exp Bot 2006 57 589 598 10.1093/jxb/erj043 16410260

[B67] Bagnoli F Danti S Magherini V Cozza R Innocenti AM Racchi ML Molecular cloning, characterization and expression of two catalase genes from peach Funct Plant Biol 2004 31 349 357 10.1071/FP03203 32688905

[B68] Wu Z Burns JK A beta-galactosidase gene is expressed during mature fruit abscission of 'Valencia' orange (*Citrus sinensis*) J Exp Bot 2004 55 1483 1490 10.1093/jxb/erh163 15208347

[B69] Dal Cin V Danesin M Boschetti A Dorigoni A Ramina A Ethylene biosynthesis and perception in apple fruitlet abscision (*Malus domestica *L. Borck) J Exp Bot 2005 56 2995 3005 10.1093/jxb/eri296 16203755

[B70] Chaves ALS de Mello-Farias PC Ethylene and fruit ripening: From illumination gas to the control of gene expression, more than a century of discoveries Genet Mol Biol 2006 29 508 515

[B71] Tieman D Taylor M Schauer N Fernie AR Hanson AD Klee HJ Tomato aromatic amino acid decarboxylases participate in synthesis of the flavor volatiles 2-phenylethanol and 2-phenylacetaldehyde Proc Natl Acad Sci USA 2006 103 8287 8292 10.1073/pnas.0602469103 16698923 PMC1472464

[B72] Dirlewanger E Cosson P Tavaud M Aranzana MJ Poizat C Zanetto A Arús P Laigret R Development of microsatellite markers in peach (*Prunus persica *(L.) Batsch) and their use in genetic diversity analysis in peach and sweet cherry (*Prunus avium *L.) Theor Appl Genet 2002 105 127 138 10.1007/s00122-002-0867-7 12582570

[B73] Aranzana MJ Garcia-Mas J Carbo J Arús P Development and variability analysis of microsatellite markers in peach Plant Breed 2002 121 87 92 10.1046/j.1439-0523.2002.00656.x

[B74] Sosinski B Gannavarapu M Hager LD Beck LE King GJ Ryder CD Rajapakse S Baird WV Ballard RE Abbott AG Characterization of microsatellite markers in peach (*Prunus persica *(L.) Batsch) Theor Appl Genet 2000 101 421 428 10.1007/s001220051499

[B75] Cantini C Iezzoni AF Lamboy WF Boritzki M Struss D DNA fingerprinting of tetraploid cherry germplasm using simple sequence repeats J Am Soc Hort Sci 2001 126 205 209

[B76] Struss D Ahmad R Southwick SM Boritzki M Analysis of sweet cherry (*Prunus avium *L.) cultivars using SSR and AFLP markers J Am Soc Hort Sci 2003 128 904 909

[B77] Cipriani G Lot G Huang WG Marrazzo MT Peterlunger E Testolin R AC/GT and AG/CT microsatellite repeats in peach (Prunus persica (L.) Batsch): isolation, characterization and cross-species amplification in *Prunus* Theor Appl Genet 1999 99 65 72 10.1007/s001220051209

[B78] Testolin R Marrazzo T Cipriani G Quarta R Verde I Dettori MT Pancaldi M Sansavini S Microsatellite DNA in peach (*Prunus persica *L. Batch) and its use in fingerprinting and testing the genetic origin of cultivars Genome 2000 43 512 520 10.1139/gen-43-3-512 10902716

[B79] Testolin R Messina R Lain O Marrazzo MT Huang WG Cipriani G Microsatellites isolated in almond from an AC-repeat enriched library Mol Ecol Notes 2004 4 459 461 10.1111/j.1471-8286.2004.00700.x

[B80] Waldron J Peace CP Searle IR Furtado A Wade N Graham MW Carroll BJ Randomly Amplified DNA Fingerprinting: A culmination of DNA marker technologies based on arbitrarily-primed PCR amplification J Biomed Biotechnol 2002 2 141 150 10.1155/S1110724302206026 12488579 PMC161367

[B81] Michelmore RW Paran I Kesseli RV Identification of markers lined to diseaseresistance genes by bulked segregant analysis: A rapid method to detect markers in specific genomic regions by using segregating populations Proc Natl Acad Sci USA 1991 88 9828 9832 10.1073/pnas.88.21.9828 1682921 PMC52814

[B82] Ahmad R Potter D Southwick SM Genotyping of peach and nectarine cultivars with SSR and SRAP molecular markers J Amer Soc Hort Sci 2004 129 204 210

[B83] Rozen S Skaletsky HJ Krawetz, S, Misener S Primer3 on the WWW for general users and for biologist programmers Bioinformatics Methods and Protocols: Methods in Molecular Biology 2000 Totowa: Humana Press 365 386 10.1385/1-59259-192-2:365 10547847

[B84] Van Ooijen JW JoinMap^® ^4, Software for the calculation of genetic linkage maps in experimental populations 2006 Kyazma B.V., Wageningen, Netherlands

[B85] Kosambi DD The estimation of map distance from recombination values Ann Eugen 1944 12 172 175

