# Supplementary material for: A fruit quality gene map of Prunus
Source: BMC Genomics. 2009 Dec 8;10:587. doi: 10.1186/1471-2164-10-587 (PMC2797820; doi:10.1186/1471-2164-10-587)
Supplement: Additional file 1 — Table S1- Features of dominant amplicons (accessory markers) generated alongside the target PCR products of candidate and cold responsive genes mapped to Pop-DG. The data provided represent information on accession number, map location, and fragment size information of dominant amplicons (accessory markers) generated alongside the target PCR products of candidate and cold responsive genes mapped to Pop-DG. [file 1471-2164-10-587-S1.DOC]

**Additional file 1 - TableS1** Features of dominant amplicons (accessory markers) generated alongside the target PCR products of candidate and cold responsive genes mapped to Pop-DG

| **LG** | **Marker code** | **CG/CRG^a^** | **Clone/Accession #** | CG type^b^ | Size (bp)^c^ |
| --- | --- | --- | --- | --- | --- |
| 1 | *aco1-c* | ACO1 | BU039036 | Texture | 450 |
|  | *epg2-c* | endoPG | BU040689 | Texture | 400 |
|  | *pme5-a* | PME5 | BU044844 | Texture | 700 |
|  | *pme5-b* | PME5 | BU044844 | Texture | 650 |
|  | *sagt-d* | SAGT | CZ445421 | *Other* | 760 |
| 2 | *aco1-a* | ACO1 | BU039036 | Texture | 630 |
|  | *aco1-b* | ACO1 | BU039036 | Texture | 500 |
|  | *epg2-b* | endoPG | BU040689 | Texture | 300 |
|  | *rin-c* | RIN | BU045116 | Texture | 350 |
|  | *sagt-c* | SAGT | CZ445421 | *Other* | 395 |
| 4 | *rin-a* | RIN | BU045116 | Texture | 430 |
|  | *rin-b* | RIN | BU045116 | Texture | 375 |
| 5 | *sagt-b* | SAGT | CZ445421 | *Other* | 570 |
| 6 | *aco1-d* | ACO1 | BU039036 | Texture | 280 |
|  | *epg1-b* | endoPG | BU040689 | Texture | 200 |
| 8 | *epg2-a* | endoPG | BU040689 | Texture | 805 |
|  | *sagt-a* | SAGT | CZ445421 | *Other* | 595 |

^a^: candidate and cold responsive genes from which the accessory markers were generated

^b^: *other* = candidate genes for putative roles other than fruit ripening

^c^: approximate amplicon size (bp) observed on polyacrylamide gel.
