# Supplementary material for: A fruit quality gene map of Prunus
Source: BMC Genomics. 2009 Dec 8;10:587. doi: 10.1186/1471-2164-10-587 (PMC2797820; doi:10.1186/1471-2164-10-587)
Supplement: Additional file 2 — Table S2 - Characteristics of 71 new Prunus ChillPeach EST-SSR markers mapped to the peach/Prunus genome. The data provided represent information on unigene, map location, and functional annotation of 71 new Prunus ChillPeach EST-SSR markers mapped to the peach/Prunus genome. [file 1471-2164-10-587-S2.DOC]

**Additional file 2 - TableS2** Characteristics of 71 new *Prunus* ChillPeach EST-SSR markers mapped to the peach/*Prunus* genome

| **Marker code** | **Unigene** | **Bin/LG^a^** | **Functional Annotation** |
| --- | --- | --- | --- |
|  |  |  |
| ***Mapped to Pop-DG*** | | | |
| C-PP04A01 | PP1004A01-T7_c_s | 1 | Biotin synthase |
| C-PPN09C01 | PPN009C01-T7_c_s | 1 | No annotation available |
| C-PPN28F07 | PPN028F07-T7_c_s | 1 | Protein At5g28080 |
| C-PPN42H06 | PPN042H06-T7_c_s | 1 | No annotation available |
| C-1128 | CL1128Contig1 | 2 | POZ/BTB containing-protein AtPOB1 |
| C-PPN70C09-B | PPN070C09-T7_c_s | 3 | Farnesyltransferase beta subunit |
| C-PPN70C09-C | PPN070C09-T7_c_s | 3 | Farnesyltransferase beta subunit |
| C-PPN18B09 | PPN018B09-T7_c_s | 4 | No annotation available |
| C-PPN37C09 | PPN037C09-T7_c_s | 4 | Putative pRGR1 |
| C-PPN46E08 | PPN046E08-T7_c_s | 4 | AJ823395 S4 *Prunus* *persica* cDNA clone S412C9, mRNA sequence |
| C-PPN61D11 | PPN061D11-T7_c_s | 4 | Transfactor-like |
| C-PPN70A04 | PPN070A04-T7_c_s | 4 | F17F8.14 |
| C-0402 | CL402Contig1 | 5 | T12C24.13 |
| C-1431 | CL1431Contig1 | 5 | Zinc finger (C3HC4-type RING finger) family protein |
| C-PPN24D05-A | PPN024D05-T7_c_s | 5 | F2D10.15 |
| C-PPN24D05-B | PPN024D05-T7_c_s | 5 | F2D10.15 |
| C-0593 | CL593Contig1 | 6 | Zinc finger, RING-type; RINGv |
| C-PP05H01 | PP1005H01-T7_c_s | 6 | Similar to Lysine ketoglutarate reductase trans-splicing related 1-like *Oryza* *sativa*) |
| C-PPN69B01 | PPN069B01-T7_c_s | 6 | TRNA intron endonuclease, catalytic C-terminal |
| C-PPN70C09-A | PPN070C09-T7_c_s | 6 | Farnesyltransferase beta subunit |
| C-0181 | CL181Contig1 | 7 | Thioredoxin domain 2; Thioredoxin fold |
|  | | | |
| ***Bin-mapped to TxE*** | | | |
| C-0139 | CL139Contig1 | 1:34 | Ubiquitin; Seven in absentia protein |
| C-0472 | CL472Contig1 | 1:50 | 6-phosphogluconolactonase |
| C-0511 | CL511Contig1 | 1:50 | *Malus* x *domestica* transcript; similar to CTV.22 [*Poncirus trifoliata* (Hardy orange)] |
| C-PPN36E09 | PPN036E09-T7_c_s | 1:50 | No annotation available |
| C-PPN73A08 | PPN073A08-T7_c_s | 1:50 | AT4g39670/T19P19_60 |
| C-PPN59H04 | PPN059H04-T7_c_s | 1:73 | Protein At5g28830 |
| C-0449 | CL449Contig1 | 1:73 | T12C22.5 protein |
| C-PPN11D11 | PPN011D11-T7_c_s | 1:73 | At5g25752 |
| C-PPN40B08 | PPN040B08-T7_c_s | 2:08 | No annotation available |
| C-0301 | CL301Contig1 | 2:45 | Membrane protein, putative; 61952-60281 |
| C-PPN51C10 | PPN051C10-T7_c_s | 2:45 | ABI3-interacting protein 2, AIP2 |
| C-PPN71B02 | PPN071B02-T7_c_s | 2:45 | Chlorophyll a/b-binding protein |
| C-1294 | CL1294Contig1 | 2:50 | Pm52 protein |
| C-PP01A03 | PP1001A03-T7_c_s | 3:14 | Protein phosphatase 2C-like |
| C-PPN40G11 | PPN040G11-T7_c_s | 3:14 | F6D8.18 protein |
| C-PPN14A03 | PPN014A03-T7_c_s | 3:36 | Ankyrin |
| C-PPN58D07 | PPN058D07-T7_c_s | 3:36 | Endoribonuclease |
| C-PPN65H09 | PPN065H09-T7_c_s | 3:36 | Hexaprenyldihydroxybenzoate methyltransferase, mitochondrial precursor |
| C-0663 | CL663Contig1 | 3:49 | Emb|CAB62340.1 |
| C-1116 | CL1116Contig1 | 3:49 | CXE carboxylesterase |
| C-PP01E04 | PP1001E04-T7_c_s | 3:49 | MYB transcription factor MYB60 |
| C-0212 | CL212Contig1 | 4:18 | Acetyl Co-A acetyltransferase |
| C-PPN52H08 | PPN052H08-T7_c_s | 4:22 | Zinc finger, RING-type |
| C-1077 | CL1077Contig1 | 4:27 | Arabidopsis thaliana genomic DNA, chromosome 5, TAC clone:K15C23 |
| C-PP02B06 | PP1002B06-T7_c_s | 4:63 | PP_YEb0038G04 Peach developing fruit mesocarp Stage S4 *Prunus persica* cDNA clone |
| C-PP03C02 | PP1003C02-T7_c_s | 5:46 | AT3g18370/MYF24_8 |
| C-PPN05E11 | PPN005E11-T7_c_s | 5:49 | SufE-like protein, chloroplast precursor |
| C-1399 | CL1399Contig1 | 6:00 | Zinc finger, RING-type |
| C-0503 | CL503Contig1 | 6:25 | Vitis vinifera transcript; similar to Serine/threonine kinase [*Persea americana* (Avocado)] |
| C-PPN07A01 | PPN007A01-T7_c_s | 6:25 | UPI0000196CCB; protein binding / zinc ion binding |
| C-PPN52F05 | PPN052F05-T7_c_s | 6:25 | Gibberellin 3-beta hydroxylase |
| C-PP02D11 | PP1002D11-T7_c_s | 6:39 | Rac GTPase activating protein |
| C-PPN28E06 | PPN028E06-T7_c_s | 6:39 | Protein kinase |
| C-PPN58D08 | PPN058D08-T7_c_s | 6:45 | *Populus trichocarpa* transcript; similar to T3H13.6 protein |
| C-PPN33E02 | PPN033E02-T7_c_s | 6:65 | Chloroplast lumen common protein family |
| C-0328 | CL328Contig1 | 6:65 | Putative oxysterol-binding protein |
| C-1182 | CL1182Contig1 | 6:80 | BZIP transcription factor bZIP105 |
| C-1290 | CL1290Contig1 | 6:84 | T6K12.3 protein |
| C-PPN31E06 | PPN031E06-T7_c_s | 6:84 | Glutamine-fructose-6-phosphate transaminase 2 |
| C-PPN12C07 | PPN012C07-T7_c_s | 6:84 | F7O18.6 protein |
| C-PPN19B01 | PPN019B01-T7_c_s | 6:84 | No annotation available |
| C-1358 | CL1358Contig1 | 7:25 | T16E15.10 protein |
| C-0838 | CL838Contig1 | 7:41 | No annotation available |
| C-PPN26E05 | PPN026E05-T7_c_s | 7:71 | No annotation available |
| C-PPN54H03 | PPN054H03-T7_c_s | 7:71 | Putative bHLH transcription factor |
| C-PPN80B12 | PPN080B12-T7_c_s | 7:71 | Ubiquitin family protein, contains INTERPRO:IPR000626 ubiquitin domain |
| C-PPN38D05 | PPN038D05-T7_c_s | 8:11 | No annotation available |
| C-PPN30D04 | PPN030D04-T7_c_s | 8:19 | Heat shock protein DnaJ, N-terminal |
| C-PPN20B08 | PPN020B08-T7_c_s | 8:30 | Dbj|BAA84809.1 |
| C-PP03H01 | PP1003H01-T7_c_s | 8:60 | Gb|AAC14054.1 |
|  |  |  |  |
| ***Monomorphic in both Pop-DG and TxE*** | | | |
| C-0219 | CL219Contig1 |  | Calcium-binding EF-hand |
| C-0396 | CL396Contig1 |  | MATH domain, putative |
| C-PPN13C06 | PPN013C06-T7_c_s |  | Membrane associated protein |
| C-PPN20F01 | PPN020F01-T7_c_s |  | PP_LEa0021I17f Peach developing fruit mesocarp *Prunus persica* cDNA clone |
| C-PPN27F07 | PPN027F07-T7_c_s |  | Protein At1g07440 |
| C-PPN32G10 | PPN032G10-T7_c_s |  | No annotation available |
| C-PPN58E02 | PPN058E02-T7_c_s |  | F7H2.8 protein |
| C-PPN40H02 | PPN040H02-T7_c_s |  | Type-B response regulator |

^a^: Linkage group of Pop-DG or bin location of T×E Pop-DG
