# Supplementary material for: A fruit quality gene map of Prunus
Source: BMC Genomics. 2009 Dec 8;10:587. doi: 10.1186/1471-2164-10-587 (PMC2797820; doi:10.1186/1471-2164-10-587)
Supplement: Additional file 3 — Table S3 - Characteristics of candidate genes (CGs) bin-mapped to the T × E reference Prunus map. The data provided represent information on the genome location (bin name), marker code, clone/accession number, source of ESTs, and CG class of all CGs bin-mapped to the T × E reference Prunus map. [file 1471-2164-10-587-S3.DOC]

**Additional File 3 TableS3:** Characteristics of candidate genes (CGs) bin-mapped to the T×E reference *Prunus* map

| **Bin name** | **Marker code** | **Functional Annotation** | **Clone/Accession #** | **EST Source** | **CG type^a^** |
| --- | --- | --- | --- | --- | --- |
|  |  |  |  |
| 1:14 | PME3 | Putative pectin esterase 2.1 precursor | BU043446 | GDR | Texture |
|  | Ara2 | Alpha-L-arabinofuranosidase / beta-D-xylosidase related cluster | CL801Contig1 | ChillPeach | CIRG |
|  | CIPK1 | Protein kinase; NAF related cluster; calcineurin B-like protein-interacting protein kinase | PPN080C05-T7_c_s | ChillPeach | CIRG |
|  | Unk8 | No annotation available | PPN046H04-T7_c_s | ChillPeach | CIRG |
|  | PDK2 | Mitochondrial pyruvate dehydrogenase kinase isoform 2 related cluster | CL142Contig1 | ChillPeach | CIRG |
|  | RGA3 | Eukaryotic translation initiation factor iso4E related cluster | PPN063E11-T7_c_s | ChillPeach | Other |
|  | C-CoAR | Cinnamoyl-CoA reductase-like protein related cluster | CL362Contig1 | ChillPeach | CIRG |
| 1:26 | Unk6 | No annotation available | PPN048E11-T7_c_s | ChillPeach | CIRG |
|  | Polyub | Polyubiquitin related cluster | CL28Contig1 | ChillPeach | CIRG |
| 1:28 | Unk15 | No annotation available | CL39Contig1 | ChillPeach | CIRG |
| 1:34 | PCCAO | Peroxisomal-copper-containing-amine-oxidase | Contig7656 | GDR | Pigment |
|  | FAH1 | Putative ripening-related P-450 enzyme related cluster | CL130Contig1 | ChillPeach | Texture |
|  | Unk25 | Unknown | PPN017G08-T7_c_s | ChillPeach | CIRG |
| 1:50 | SAMDC1 | S-adenosylmethionine decarboxylase | Contig7174 | GDR | Texture |
|  | 4CL | 4-coumarate-CoA ligase-like protein related cluster | CL694Contig1 | ChillPeach | *Other* |
|  | Unk29 | No annotation available | CL852Contig1 | ChillPeach | CIRG |
|  | AspAT2 | Putative aspartate aminotransferase related cluster | CL149Contig1 | ChillPeach | CIRG |
| 1:52 | ACO2 | 1-aminocyclopropane-1-carboxylate oxidase - like protein | BU041281 | GDR | Texture |
| 1:55 | Chit1b | Chitinase Ib related cluster | CL698Contig1 | ChillPeach | CIRG |
|  | Unk9 | No annotation available | PP1000E03-T7_c_s | ChillPeach | CIRG |
|  | SREB | Sucrose responsive element binding protein related cluster | CL757Contig1 | ChillPeach | CIRG |
| 1:73 | COMT | Phloroglucinol O-methyltransferase related cluster | PPN021G12-T7_c_s | ChillPeach | Other |
| 1:87 | CAP | Cytosolic ascorbate peroxidase related cluster | CL6Contig1 | ChillPeach | CIRG |
| 2:08 | Pel3 | Pectate lyase | BU045635 | GDR | Texture |
|  | Gal2 | Beta galactosidase-like protein | BU040993 | GDR | Texture |
|  | Exp4 | Expansin | AJ533090 | Trainotti et al. 2003 | Texture |
|  | ERP | Ethylene-responsive small gtp-binding protein | BU048565 | GDR | Texture |
|  | NXCE | Neoxanthin-cleavage-enzyme |  | GDR | Pigment |
| 2:13 | O-6FAD | Omega-6 fatty acid desaturase related cluster | CL1Contig1 | ChillPeach | CIRG |
| 2:25 | Unk3 | Unknown protein | AJ533607 | Trainotti & Casadoro (unpublished) | Texture |
|  | RHB1 | RING-H2 finger protein RHB1a related cluster | PPN077H09-T7_c_s | ChillPeach | CIRG |
|  | Thioest | T1N6.10 protein related cluster /acyl-CoA thioesterase/ cyclic nucleotide binding | PPN049G01-T7 | ChillPeach | *Other* |
| 2:28 | ACS1 | 1-aminocyclopropane-1-carboxylate synthase 1 (acc synthase) (s-adenosyl-l-methionine methylthioadenosine-lyase) | BU041005 | GDR | Texture |
|  | CP | Cysteine proteinase related | CL81Contig1 | ChillPeach | CIRG |
|  | ACS2 | 1-aminocyclopropane-1-carboxylate synthase | BU047017 | GDR | Texture |
| 2:34 | PME4 | Ripening-related protein-like; contains similarity to pectinesterase | BU046426 | GDR | Texture |
| 2:38 | PMIP | Plasma membrane intrinsic protein related cluster | CL3Contig1 | ChillPeach | CIRG |
| 2:45 | Unk1 | Unknown protein | BU040337 | GDR | Texture |
|  | CrtL | Highly similar to Lycopene beta-cyclase related cluster | CL197Contig1 | ChillPeach | Pigment |
|  | GDH | Glutamate dehydrogenase 2 related cluster | PP1004F04-T7_c_s | ChillPeach | CIRG |
|  | SDH | Sorbitol dehydrogenase related cluster | PP1003C10-T7_c_s | ChillPeach | Flavor |
| 2:50 | TTG1 | Similar to Ttg1-like protein homolog | DY635300 | GDR | Other |
| 3:04 | Exp2 | Expansin | BU041271 | GDR | Texture |
|  | Exp3 | Expansin | BU046518 | GDR | Texture |
|  | SDH | Sorbitol dehydrogenase related cluster | PP1003C10-T7_c_s | ChillPeach | Flavor |
| 3:06 | PRS12 | Proteasome regulatory subunit S12 | CL576Contig1 | ChillPeach | CIRG |
|  | PSY | Phytoene synthase-like | CL305Contig1 | ChillPeach | Pigment |
|  | Unk7 | No annotation available | PPN049D06-T7_c_s | ChillPeac | CIRG |
|  | ICDH | NADP-dependent isocitrate dehydrogenase related cluster | PP1001B08-T7_c_s | ChillPeach | Flavor |
|  | Unk30 | Related to UPI00004C154C; phytanoyl-CoA dioxygenase domain containing 1 | CL483Contig1 | ChillPeach | CIRG |
| 3:12 | EREB | Ethylene responsive element binding protein | PP_LEa0004J07.b1 | GDR | Texture |
|  | PSA6 | Proteasome subunit alpha type 6 related cluster | CL2Contig1 | ChillPeach | CIRG |
|  | Stress | Abscisic stress ripening-like protein | BU043120 | GDR | Texture |
| 3:14 | RRP | Putative ripening-related protein related cluster | CL86Contig2 | ChillPeach | CIRG |
| 3:22 | PG3 | Polygalacturonase - like protein | BU043904 | GDR | Texture |
|  | EGase | Endo-beta-1,4-glucanase, putative | BU048008 | GDR | Texture |
|  | PEPC | Phosphoenolpyruvate carboxylase related cluster | CL1222Contig1 | ChillPeach | Flavor |
|  | LitP | Little protein 1 related cluster | PPN043F06-T7_c_s | ChillPeach | CIRG |
|  | PGDH | D-3-phosphoglycerate dehydrogenase | PP1003B08-T7_c_s | ChillPeach | CIRG |
|  | Unk2 | Unknown protein | BU041661 | GDR | Pigment |
| 3:36 | PP2C | Protein phosphatase 2C-like related cluster | PPN054D04-T7_c_s | ChillPeach | CIRG |
|  | Unk27 | Unknown (highly similar to YUP8H12.27 protein related cluster) | PPN017G10-T7_c_s | ChillPeach | CIRG |
|  | RGA4 | Potyvirus VPg interacting protein related cluster | PPN057E11-T7_c_s | GDR | *Other* |
|  | AXR1 | Putative auxin-resistance protein related cluster | PPN078G01-T7_c_s | ChillPeach | *Other* |
|  | ACO1 | 1-aminocyclopropane-1-carboxylate oxidase | BU039036 | GDR | Texture |
| 3:49 | PME2 | Putative pectinesterase | BU043756 | GDR | Texture |
|  | DPO | Diphenoloxidase | PP_LEa0007G20 | GDR | Pigment |
|  | AGRT | Anthocyanidin-3-glucoside rhamnosyltransferase | BU039808 | GDR | Pigment |
| 4:18 | ACO3 | 1-aminocyclopropane-1-carboxylate oxidase gene family | AF129074 | Ruperti et al., 2001 | Texture |
| 4:27 | Unk17 | No annotation available | PPN026F04-T7_c_s | ChillPeach | CIRG |
| 4:28 | TAT | Putative tyrosine aminotransferase related cluster | PPN018G03-T7_c_s | Chillpeach | CIRG |
|  | Unk28 | No annotation available | PPN030H04-T7_c_s | ChillPeach | CIRG |
| 4:46 | SAMM-a | S-adenosylmethionine:2-demethylmenaquinone methyltransferase-like protein | BU047583 | GDR | Texture |
|  | GPPDE | Putative glycerophosphoryl diester phosphodiesterase family protein related cluster | PPN046G07-T7 | ChillPeach | *Other* |
|  | SeCy | Sesquiterpene cyclase related cluster | PPN001B06-T7_c_s | ChillPeach | CIRG |
| 4:63 | Gal1 | Beta-galactosidase (emb|cab64746.1) | BU043071 | GDR | Texture |
|  | PK | Protein kinase related cluster | CL550Contig1 | ChillPeach | CIRG |
| 5:04 | PAE1 | Pectin acetylesterase (ec 3.1.1.-) precursor | BU039972 | GDR | Texture |
|  | PAE2 | Pectin acetylesterase (ec 3.1.1.-) precursor | BU039692 | GDR | Texture |
|  | Cat1 | *Prunus persica* mRNA for catalase (cat1 gene) | AJ496418 | Bagnoli et al., 2004 | Texture |
| 5:21 | SAGTb | Protein At2g43840 | PPN069B11-T7_c_s | ChillPeach | *Other* |
| 5:41 | GluRed | Glutaredoxin, eukaryotic and viruses related cluster | PPN048A04-T7_c_s | ChillPeach | CIRG |
| 5:46 | Pel1 | Pectate lyase | BU039491 | GDR | Texture |
| 6:25 | Exp1 | Expansin 1 | PP_LEa0003N18 | GDR | Texture |
|  | Exp3 | Expansin | PP_LEa0030G20 | GDR | Texture |
|  | SAMM-b | S-adenosylmethionine:2-demethylmenaquinone methyltransferase-like protein | BU047583 | GDR | Texture |
|  | R-Zinc | Highly similar to Putative RING zinc finger ankyrin protein related cluster | CL1011Contig1 | ChillPeach | CIRG |
| 6:39 | CS | Capsanthin/capsorubin synthase | CL197Contig1 | ChillPeach | Pigment |
| 6:65 | Pel4 | Putative pectate lyase | BU043362 | GDR | Texture |
|  | PGIP | Highly similar to Polygalacturonase-inhibiting protein related cluster | CL146Contig1 | ChillPeach | CIRG |
|  | PAL | Phenylalanine | Prunus_v3_Contig731 | GDR | Pigment |
| 6:74 | AGAT | Alanine--glyoxylate aminotransferase 2 homolog 2, mitochondrial precursor related cluster | CL1351Contig1 | ChillPeach | CIRG |
|  | CDTPL | C-terminal domain phosphatase-like 2 related cluster | PPN070H08-T7_c_s | ChillPeach | CIRG |
| 6:80 | Unk15 | No annotation available | CL39Contig1 | ChillPeach | CIRG |
| 6:84 | Sod4a | Superoxide dismutase | CL1376Contig1 | ChillPeach | *Other* |
|  | Unk24 | No annotation available | PPN069H05-T7_c_s | ChillPeach | CIRG |
| 7:25 | LycB | Lycopene-beta-cyclase | PP_LEa0025L06 | GDR | Pigment |
|  | SIP | Putative seed imbibition protein related cluster | CL1468Contig1 | ChillPeach | CIRG |
| 7:31 | CWI | Highly similar to 40S ribosomal protein S14-3 related cluster | PPN045D11-T7_c_s | ChillPeach | Flavor |
| 7:41 | PG2 | Polygalacturonase | BU047552 | GDR | Texture |
|  | Gal3 | Beta-galactosidase | AJ278703 | Trainotti et al. 2003 | Texture |
|  | UFGT | Anthocyanidin 3-O-glucosyltransferase related cluster | PPN007E12-T7_c_s | ChillPeach | CIRG |
|  | Dehy2 | Dehydrin 2 related cluster | CL85Contig1 | ChillPeach | CIRG |
| 7:48 | Unk17 | Unknown (highly similar to YUP8H12R.13 protein related cluster) | PPN026F04-T7_c_s | ChillPeach | CIRG |
| 7:56 | AspS | Asparagine synthetase related cluster | CL283Contig1 | ChillPeach | CIRG |
| 7:71 | GT | glucosyltransferase | AJ533938 | Trainotti et al. 2003 | Texture |
|  | SAGTa | Protein At2g43840 | PPN069B11-T7_c_s | ChillPeach | *Other* |
| 8:11 | PME6 | Pectinesterase, putative | BU039266 | GDR | Texture |
|  | AADC1A | Highly similar to Putative serine decarboxylase related cluster | CL1274Contig1 | ChillPeach | Flavor |
| 8:60 | ER2 | Ethylene receptor (ERS1) | AY061640 | Bonghi et al (unpublished) | Texture |
|  | PKS1 | Highly similar to Chalcone synthase 2 related cluster | CL792Contig1 | ChillPeach | CIRG |
|  | Ribo2 | Highly similar to Ribonuclease 2 precursor related cluster | PPN014H05-T7_c_s | ChillPeach | CIRG |

^a^: CIRG = chilling injury resistance genes; *other* = candidate genes for other putative roles such as resistance, hairiness, cutin and lignin synthesis.
