# Supplementary material for: A fruit quality gene map of Prunus
Source: BMC Genomics. 2009 Dec 8;10:587. doi: 10.1186/1471-2164-10-587 (PMC2797820; doi:10.1186/1471-2164-10-587)
Supplement: Additional file 4 — Table S4 - Primer details for candidate genes and EST-SSRs. The data provided represent information on the primer sequence, annealing temperature, amplicons size, and type of polymorphisms for candidate genes and EST-SSRs. [file 1471-2164-10-587-S4.DOC]

Additional File 4 – TableS4: Primer details for candidate genes and EST-SSRs

| **Primer Name** | **Forward (5’ – 3’)** | **Reverse (5’ – 3’)** | **Ann Temp (^0^C)** | **Amplicon Size^a^** | **Polymorphism type^b^** |
| --- | --- | --- | --- | --- | --- |
| 4CL | CCCTCAAATTGTTGATGCTG | GCTATGAATGCCACTCTCCTG | 60 | 174 | Indel/SSCP |
| AADC1A | GCAGCGAGAATGTATAGAATGG | CCGTCGCAGTGAATGTAGAA | 60 | 230 | SSCP |
| ABA-IP | GCTAGCTGCTCATCATGATCTC | ATAGGCAGAGCCCACTTTCC | 57 | 235 (500) | Indel |
| ACO1 | AACAACACTTGTTGAGAGAG | AGTGTTCTTTTGTCAACCTC | 57 | 183 | Indel |
| ACO2 | GTGCCCTCTCTATTGCTTGG | TGCTGCTTCAGGACCACAT | 60 | 223 (320) | SSCP |
| ACO3 | TCTTCAGGGTCAGTCAGGGTA | TTTATAGGCGCGAAGGGAGT | 60 | 241 | Inndel |
| ACS1 | GATGGTGAGATGGTGCTTTG | TCTCATTTAAACTGACCACCCTTA | 60 | 214 | SSCP |
| ACS2 | CGACTGCTGCCATTGAGAT | GGAGTAGATAACACCCGCCTTA | 60 | 580 (488) | SSCP |
| AGAT | GGGCAACTGGAAATTCAATG | GCCTCCTGCTTTCTTCACAC | 60 | 247 (1500) | Indel |
| AGRT | ACAgAgggTgggAgAgAAgg | CCCTCTACAAATTTATCCACAACC | 60 | 300 | SSCP |
| Aqp | TTGCACGCCACAACCCAC | GCCTTCATCTTGGGTCTGAGC | 60 | 224 | Indel |
| Ara | CAACCAAGGCAAGAAGGAAG | AAGACAGACCCTTTACCAGCA | 60 | 400 (247) | Indel/SSCP |
| Ara2 | TGAAGCCGAATTCGTAGACC | AACATCGGCTATGGCAGTTC | 60 | 217 | SSCP |
| ARG2 | GCCGATGGATTCTCTAGCAC | ATTCTCGGGTCCGTAAAACC | 60 | 204 (360) | Indel |
| AspAT1 | TCAGCAAATCGATACCTGGTC | ATCTTCGGACATGGATGAGC | 60 | 245 | SSCP |
| AspAT2 | CGGTTGTTAATCCTGGCAAC | CCATCCCATCATCCCATAAG | 60 | 212 (1200) | SSCP |
| AspS | TCTTGACCCATCTGGTAGGG | CACTAAGGCATCGCAAAGTC | 60 | 246 | SSCP |
| Aux-IAA | GCCGTATGGAGGGTCCTTAG | GCCTTCAGTTCCTTCCCTTC | 60 | 223 (400) | Indel |
| AXR1 | GCAGAGGAGTGGTCCAAAAG | CCATCACCCAAAAATCAGATG | 60 | 238 (700) | SSCP |
| BCH | CAATTCAACCCACCCTCAAT | GAGCTTCCTTCGAGCCCTAT | 60 | 210 | Indel |
| C-0139 | CAATCGCAACAACAGCTACG | ACTTGCGGTAAGCAGCCAAC | 57 | 176 | SSR |
| C-0181 | TCAGCTGCTGTGATGTTCTTG | ACATTGCTGGTTTCCTTTCC | 57 | 160 | SSR |
| C-0212 | AACGCTCTCAAAAACTTCATCC | TGCAAACATCTCGAGGTTTAATAG | 57 | 250 | SSR |
| C-0219 | CAGCCCAGCCTAAACGTAAG | TGTAGGTCCCTTTGGTTTGC | 57 | 152 | SSR |
| C-0301 | TTCCACCAACTCCATCTGAAC | GGCCTTGCTCTGTCTTTCAC | 57 | 190 | SSR |
| C-0328 | CACCCTCGTCAAGTCTCTCTC | GAGCCATCTCCGATGTAAGC | 57 | 163 | SSR |
| C-0396 | CTCTCTGCTGCTCCTTCGAC | TCAACCTTGCTCACTGCAAC | 57 | 154 (500) | NP |
| C-0402 | AGAGAGAGTTAAACACCATTAAAA | CCTGGGCCTATCAGAGAGTG | 55 | 197 (320) | SSR |
| C-0449 | CAGGGAGAGAGAGGAAGCTC | CGCATAGCATCCTTAATCTGG | 57 | 179 | SSR |
| C-0472 | CCATCTTCCACCTTCGTCAG | TCTTTGTGACCTCCGGAAAG | 57 | 150 | SSR |
| C-0503 | TGAGCACCAACTTTGCTCTC | GGCACATCTTTCAGCATTCC | 57 | 231 (620) | SSR |
| C-0511 | CCCAACAGCCAATATTACCC | GGCTGATGCATATTTGAAAGG | 57 | 238 (780) | SSR |
| C-0593 | CATTGATCCAATGCATGCTG | CTTCCACCTGGACTCGAAAC | 57 | 207 | SSR |
| C-0663 | AGAGAGAAAGAGAGAGTGAAAGAGAG | GGTTGATTATCGTCTTATCTTGC | 57 | 180 | SSR |
| C-0838 | TGGGGATTTGATGTACTGAATG | CCCAATGTTCAAATTCTTCCA | 57 | 183 | SSR |
| C-1077 | GAGTGGTGATGATGGCTTTG | TGCTGTAGAGCAAGGTGAGG | 57 | 166 | SSR |
| C-1116 | CCTCTAATTAAGTCATACGTGACC | GCGATTCTGTCAATTGTTCC | 57 | 152 | SSR |
| C-1128 | AGAGAGGAGGGGTCATCTGC | GCAGAGTATGGGGAGAGGTG | 57 | 177 | SSR |
| C-1182 | TTCAGCACCACCAGTCAATG | GTCGTTGTGGTCATGGTGAG | 57 | 181 | SSR |
| C-1290 | TGATTGAGTGAGGGGGAGAG | CACCAAGATTCACACCCAAAC | 57 | 220 | SSR |
| C-1294 | GGATTGCTAGGGTTTTAATAGGC | GGTCTCCGTGAACTGCTCTC | 57 | 155 | SSR |
| C-1358 | TCTTGTGTTGAAGTGGTGGC | CGATCCACCAATCTCCTAGC | 57 | 148 | SSR |
| C-1399 | ATGCCCTTTTGTTCATTTCC | TGTCTCGGTTGATGCCTATG | 57 | 177 | SSR |
| C-1431 | AATTCATGTCCTCTACTGTTTCTTC | TTCTGTGCTCGTCACCTTTG | 57 | 171 (250) | SSR |
| CAP | CCCCTCATCTTCGACAACTC | GCAAACCCAAGCTCAGAAAG | 60 | 197 (360) | SSCP |
| Cat1 | TCCTTTCTggAAAgCgTgAg | TCACATTgAgACgggATgC | 60 | 720 | Indel |
| C-CoAR | TTGACTGCAGCAATTTGGAG | GGAAACCATCTTGAGCATCC | 60 | 174 (600) | Indel |
| Chit1b | CTGCTTTCTTGGCTCAAACC | TCCTTTCCGATTGCATTACC | 60 | 229 (550) | SSCP |
| CIPK1 | TGCACACAAGGGAAAACTTG | AGAGGTGGCACCATCATACC | 60 | 280 (480) | SSCP |
| CND | CTCTTTGGAGAAGGGCTTTG | GACCTTTGGTGGAGGAGGAG | 57 | 122 | SSR |
| COMT | GATGGAGTTGGGTCTGTTGG | CCTGGTTGAATACCCGATTG | 60 | 181 (800) | SSCP |
| CP | CGTCAACCATGTCCCTAACC | GTTCAGGCCAACCTTGTAGG | 60 | 248 | SSCP |
| C-PP01A03 | TGTATTCCGTGGTTCGATAGC | GTGCACGGCTACTCCACAG | 57 | 224 | SSR |
| C-PP01E04 | TCTGAGGATATAGCAAGGAAGACC | TGACCCCTTCTTTATCACAGC | 57 | 179 | SSR |
| C-PP02B06 | GACGAACACGAAGCATATTG | CCCATGATCAAGCCAGTCTC | 57 | 203 (1000) | SSR |
| C-PP02D11 | CCCTTTCTTCCCTTCCAATC | GGGCGCGTAGGATAAAGAAG | 57 | 357 | SSR |
| C-PP03C02 | ATCTTCAACTTTATCCGTTTTCTC | ACTCAACAGCATCCTCAACG | 57 | 291 | SSR |
| C-PP03H01 | GAGCTGGACAATTGAGCTTG | GACATGGAATTAGAGAAAATCTC | 57 | 151 | SSR |
| C-PP04A01 | CAGGCCAGCAGCCACTCT | ACAGCAACCGACGGTCTTAG | 57 | 192 | SSR |
| C-PP05H01 | CAGAATTGGGGGAGGACTTC | TCGTTTTGTTTTACGGTGTG | 57 | 293 | SSR |
| C-PPN05E11 | TGGAACCTGAAACAACAACAAC | TAAGGAATGCCAACCGTTTC | 57 | 187 | SSR |
| C-PPN07A01 | ACCCGACGTTTCCTATTTCC | CTGCAAATCCAAACCCAGAC | 57 | 209 | SSR |
| C-PPN07E01 | GACCCCAAAAATCAAAACTATAG | ACAAACAACCTCACCACTCAG | 57 | 151 | SSR |
| C-PPN09C01 | GGAAAAGCTTCTTTTCTATTTTGC | TGGACAGAGGGAATTTGGAG | 57 | 161 | SSR |
| C-PPN11D11 | CCCGGTTTATCTCTGGAACC | CGAGGTTTGAGGAGAGGTTG | 57 | 155 | SSR |
| C-PPN12C07 | TTATTCGACACAGACGCACG | AGTGGGTATTTGGCAAGAGC | 57 | 187 | SSR |
| C-PPN13C06 | AAATTTCTAAAGCGACCAACTG | GGAGGAGGAAGACGACGAC | 57 | 221 | SSR |
| C-PPN14A03 | GTGGGAAGATCCTGACGATG | CTTGCTCCAAGAGCTGTTCC | 57 | 197 (650) | SSR |
| C-PPN18B09 | GTTGTGGTGGTGGCCATTAG | GGACAAACTCCATTCTGTCACC | 57 | 156 | SSR |
| C-PPN19B01 | CGACGAAGAAACTGTTGACG | GAAACCGCCGATGAAGAAG | 57 | 204 | SSR |
| C-PPN20B08 | GGAAATGAAACATCCAAAATCTTC | GGATCGGACAGAGGAGGAC | 57 | 205 | SSR |
| C-PPN20F01 | GGTCTTTAATTCTAGAGAGAGAG | TTGGTTTCACCTCCAATTCAG | 50 | 247 | NP |
| C-PPN24D05 | GGTTGGAATTGGAGAGAGAGAG | ATGGCGGTTGAGAAAATCAG | 57 | 220 | SSR |
| C-PPN26E05 | CACGAGGAGGAGATTCGTCT | TCTGACCCCACAAACTCACA | 57 | 217 | SSR |
| C-PPN27F07 | GACAAGACAAAATTAACAACACTTG | ACCCTGCCAATTCTTCCAC | 57 | 158 (360) | NP |
| C-PPN28E06 | AGGAAGCGAGCAAAAGAGAG | GAGTCAGGAAGTTGGGCAAG | 57 | 191 | SSR |
| C-PPN28F07 | GCATGTTAAGCTATTAGCATTGG | CAGGGGCAGTAACACCATTC | 57 | 167 | SSR |
| C-PPN30D04 | GGGAGCTGAAAAGGAAAGAG | GGTCACGTGTCGTCAGTAGC | 57 | 160 | SSR |
| C-PPN31E06 | GGTTCTCTTTCTCTCTCAATCCAG | TATTCCAAACGCCTGAGACC | 57 | 159 | SSR |
| C-PPN32G10 | ATTGGAAGAGGGACCAAAGC | TCAGACCCATACTTGCATCG | 57 | 167 | SSR |
| C-PPN33E02 | ATTGTGGTGGCTGGCTTC | CGATTGCAGACGCTCTACAG | 57 | 159 | SSR |
| C-PPN36E09 | GCATGATGAAAGATCAATATACAA | CGGTAACTGCCATTCTGTCA | 57 | 261 | SSR |
| C-PPN37C09 | AGCGGAAGCAAAACAGAAAG | TCCTCATCCACCTCCATACC | 57 | 141 | SSR |
| C-PPN38D05 | GAGAAAACATTTGTTATTGTTGCTC | GGTGAGGAGAAAGCACAAGC | 57 | 142 | SSR |
| C-PPN40B08 | TTTGATGGAAACCCAAATGAG | AACAAAGTCAGCCCCACAAC | 57 | 213 | SSR |
| C-PPN40G11 | TTTCAGTACGGCCAATTGTAG | CCGGATCTGAAGGGATTTG | 57 | 153 | SSR |
| C-PPN40H02 | GGAGAGCTTGAAGCAGAAAG | CATGCTTAAGCTTCGGTTTTG | 57 | 222 | NP |
| C-PPN42H06 | CATGAGACCCATGCAGTCC | CACTCAGTCAGCGGTAACAAAC | 57 | 172 | SSR |
| C-PPN46E08 | TTGCCGGGGAGAGCTAAG | TTCCACTCGAGCCTTTCATC | 57 | 202 | SSR |
| C-PPN51C10 | TGACAAGATGCAGGAGTTGC | AGAGTGGAGCTGCAAATGAC | 57 | 272 (500) | SSR |
| C-PPN52F05 | CAACGAACTTATATCACCACCAC | TTGGAAGACACCCCAAGTTC | 57 | 279 | SSR |
| C-PPN52H08 | GAACCTTTAACTTTTGCTCTCTC | GAAAGGTTGGTTCGTTCTGC | 57 | 234 | SSR |
| C-PPN54H03 | CAAGGGTTTATGCCTCATGC | TAACGATTCAGGGCTTGGAG | 57 | 257 | SSR |
| C-PPN58D07 | AACGCATGCCACTTGATTAC | TTTCCCACATCACCAGTGTC | 57 | 193 | SSR |
| C-PPN58D08 | CACACAACCCTCAACTCTCAAC | GATTTGGCTCTGGTTTGTGG | 57 | 291 | SSR |
| C-PPN58E02 | CTTGGAGCTTGGAATTCTGG | GATTTGGCTCTGGTTTGTGG | 57 | 163 | SSR |
| C-PPN59H04 | GAATCCCAAACATCAAAATTTC | AGGAGAGGCCGAAGAGAGAC | 57 | 211 | SSR |
| C-PPN61D11 | GTGGAGATTGGGATTTGACG | GCAAGGGTCTCCTCTGTGAC | 57 | 152 | SSR |
| C-PPN65H09 | TGAATATCGTTGCACAACAGC | TAAAGCAGGCATAAAGACACC | 57 | 194 (300) | SSR |
| C-PPN69B01 | GGGAAAGGAGATAGGTCTGG | CTGCAAGGTAGACGGAGGAG | 57 | 162 | SSR |
| C-PPN70A04 | CTCCAAAAACCCAAATACCG | CACCAAACCTTCGACGAAAC | 57 | 158 | SSR |
| C-PPN70C09 | GGGGGTTTGTGTGTGTGTG | GAAGGTTGCCAAAGAGATCG | 57 | 200 | SSR |
| C-PPN71B02 | TCATCAAACATTTCACTAGCTTC | TGGACAGTTGAGAACTCTGTCTG | 57 | 157 | SSR |
| C-PPN73A08 | GGTATGGCAATGGCAGAGTC | GGCAACTGAAGAGGATGGAG | 57 | 169 | SSR |
| C-PPN80B12 | TCGTCTCCATGTCCCTTCTC | GGCTGATGATTGTGAAGCTG | 57 | 273 (1000) | SSR |
| CrtL | AGTGTGATGGCAATTGGG | AAGTGGCTGCCCTCTAATCA | 60 | 150 | SSCP |
| CS | CACGGGTTTCTCTCCTCAAG | GCCTCAAGTGCTAGATTACCC | 60 | 152 | SSCP |
| CTDPL | CCAGTGGTTTGCTCTCACAC | ACCTTTGAGCTGCATCTTCG | 60 | 238 (480) | SSCP |
| CWI | TTAGCTGGCGATGTGGTTTC | AACAGCATTTGATGTGTAAAGCTC | 60 | 175 | Indel |
| Dehy2 | GCTCCCAGGTACTCATGACC | CACCCTATGTCCACCAGTCC | 60 | 271 | SSCP |
| DPO | GAAGAATCGCCCTGGGAAT | CAGCCACAACATCACAGTGG | 60 | 248 | Indel |
| EGase | TGGCCTTCAGAAGTACAGGAA | TCAAAGGATTCTCTCCCAACA | 60 | 850 (287) | SSCP |
| endoPG | CCTTCAACTCATTAACCTCTCTCTC | GGAAGGCTTTTGTGGAGTCA | 57 | 201, 203 | SSR |
| ER2 | GGACTTTATGCCTCCACGAA | CAATTGCTTGAGAAATAGGGAAG | 60 | 164 | Indel |
| EREB | ATgAAATggTTgAggCCAAg | ATCTCTgCAgCCCACTTACC | 60 | 1300 (181) | SSCP |
| ERP | CCCTCTTTCTCTCTCCCTCTC | CCATCTGAGAAGCGCAAAAG | 60 | 229 | Indel |
| Exp1 | CCCACCTAACTTTGCTCAGTC | TTCTTCATACAGGGCACTCTTC | 60 | 600 (151) | Indel/SSCP |
| Exp2 | GCGCAGGGTGATTTGTATATG | TCAAAGATATTAATGTGCATTTTGC | 60 | 250 | Indel |
| Exp3 | ATGGAAATCTCTACAGCCAAGG | AGTTTGGAGGGCAGAAGTTG | 60 | 180 | Indel |
| Exp4 | GCGGATCGCATTTTTGTACT | GCTGACGGGTATTTTCAGGA | 60 | 550 (208) | Indel |
| FAH1 | TTATGCCGGAGAGGTTCTTG | AACCCCATCTTCAAGCTTCC | 60 | 179 | SSCP |
| Gal1 | TGGTAGCTCGCCAATTCTTC | GTCCCATTGTTGAACCCAGT | 60 | 1500 (258) | SSCP |
| Gal2b | GATATTAAGTGGCGGTTAGCTC | TGTCAACTGTTCCCGTTTGA | 64 | 290 | Indel |
| Gal3 | TCCATTAAATCTTGGCTCTTCC | GTGCTTTCGATCCCAACATT | 60 | 181 | SSCP |
| GDH | AGCTTGGTTTCGTACGTTGG | TGCAGGAACATCGGTATGAG | 60 | 282 (1000) | SSCP |
| GLABRA2 | GGGAAGTGGAGGAAGGAGAC | TCTCGGATTTGCTCAGTGG | 60 | 226 | Indel |
| GluRed | GCAACTGCTCACTCAGCTTG | TCACTGAGAAGGGGCAGAAG | 60 | 204 (700) | Indel/SSCP |
| GPPDE | TGCTGATTACTTGCCACCTG | GAAAGAAGCCAGCAGCAATC | 60 | 176 | SSCP |
| GT1 | GTGCAGGATGTTTGGAAGGT | CCGTCACTGACAAGCCTCTA | 60 | 204 | SSCP |
| ICDH | ACCTGGGAAACTGAAATTGG | AGTGGTCATGGAAGCCTCTG | 60 | 154 (400) | SSCP |
| LitP | TTTGGAGTTTCCGTTGATCC | GAATGGCATTAGAGACCTTCG | 60 | 257 | SSCP |
| LycB | GGATGGTGCATCCTTCAACT | GCAGAATAGCCATACCAAAACA | 60 | 201 | SSCP |
| MADS1 | GCAGCAGCCATTTCTCTCTC | CTGCCACCTCAGATTCACAC | 60 | 190 (600) | SSCP |
| NXCE | TTCCAAGGATGGTTTCATGC | TCCTCCCAAGCATTAGCATT | 60 | 600 (202) | Indel |
| O-6FAD | CGGTTTTCAAGGCAATGTTC | TCATGAGTGAAAGGGGAAGC | 57 | 185 | SSCP |
| PAE1 | TCTCTGGGTGTTCAGCTGGT | AGGATGCGGGTAGGTTCTTT | 60 | 430 (207) | SSCP |
| PAE2 | CATGTGCAGTCACACTGCTG | CCCAGGCAATTTTGTGACAT | 60 | 540 (203) | SSCP |
| PAL | AGAATTGATAGCCTCCTATGGG | TTGATTGCATTGCTGGTAGC | 60 | 222 | Indel |
| PAPISP | AAGGCTGACTGCTGAGGAAG | AAGACCCGTGTCATCGTAGC | 60 | 226 | Indel/SSCP |
| PCCAO | TGGAAACATCAGGATTGGAGA | CACCAGGCTTACTATCAACTGC | 60 | 243 (292) | SSCP |
| PDK2 | CCTCCCTTATGGCTTGTCTG | TCCTCGGATTTATGCCTTTC | 60 | 218 (700) | SSCP |
| PL | TGACCTATTGCGAGGGACAT | CAGGCCGAGGTTGTAATCAT | 60 | 247 | Indel/SSCP |
| PL2 | TGTTTTTCTCTCTGCTCTCTGC | CGCCAAGAAAGCACCAGTA | 60 | 186 | SSCP |
| PL3 | TTTGCCCCTGTCTCTTTTTG | CCACAGAATCATTCACATCCT | 60 | 163 | SSCP |
| PL4 | AAGGTGGTAGAGGGCCAGAT | GGTGAATGGTAACCCGGATG | 60 | 320 (231) | SSCP |
| PEPC | TCCGAACTACCATGTTAAAGTG | CCAGCAGCAATACCCTTCAT | 60 | 159 | SSCP |
| PG2 | TTACCTTGTCCACCCTCTCG | TGTCCCATCCCATTGATTCT | 60 | 310 (189) | SSCP |
| PG3 | GTTTGCCTTTTATGCCTCCA | ACGTAAATAATGAGAATAAGGTGACA | 60 | 1000 (220) | SSCP |
| PG4 | CTGACATCCCATCACATAGCA | CATTCGCAAAACCAAACAGA | 57 | 597 | SSR |
| PGDH | AAGCGAGGAAAGGGTAGCTG | TTTCCTTCCAGGCTCACATC | 60 | 201 | SSCP |
| PGIP | CTTCCCAACCTCAACGCC | ATGCGGTTGAAGTCCAGTTT | 64 | 166 (320) | SSCP |
| PK | ATAATTTAGCCCGCCAGACC | ACCCCAAAGCTGAACACATC | 60 | 226 (1400) | SSCP |
| PKS1 | GTGACAAATCTATGATCAAGAAGC | GCAGCTTCTTTGCCGAGT | 60 | 153 | Indel |
| PME1 | AGGGCCTCCTCTGTTCTGTT | GAGATGAGGGGCAACTTGAG | 60 | 295 | Indel/SSCP |
| PME2 | TGCCGAATATGGTATTCTTAGC | TTATGGATGGGTGCATCAGA | 60 | 650 (245) | SSCP |
| PME3 | TGTTGCTCTTCGAGTTGGTG | GCCACTGAACCCTACCTGAA | 60 | 506 | SSCP |
| PME4 | GCCACATGTTTTCTCACAGC | TATTCACAATCCGGCCTCTC | 60 | 520 | SSCP |
| PME5 | AGAAGATTCTGGTCGACGTGT | CCCTATCGTCACACAACGTG | 60 | 800 (297) | Indel/SSCP |
| PME6 | TCGACCACCTAGACCCAACT | GCAGTGAGCAAAGCAGGAAT | 60 | 850 (297) | SSCP |
| PMIP | TATAATTGCACGCCACAACC | GCCTCTCCGAAAACTTGTTG | 57 | 191 | Indel/SSCP |
| Polyub | CCCTTCAATTCTCTCGAAGC | GACGCTGCTGGTCTGGAG | 60 | 167 | Indel |
| PP2C | CATCAGCAGCAAGGATTGTG | CGGAGTGATGCTCTGAAGTG | 60 | 176 | SSCP |
| PpLDOX | CTGCTGATTACATGTAAGTACTCAAGG | GTAGCCTCACTGCAAAGGTAT | 57 | 238 | SSR |
| PRS12 | ATGTCCAACCAAAGGAGCTG | TCGAAGTCTCGCTTCCAATC | 60 | 248 (680) | SSCP |
| PSA6 | TGCTTATATGAGACCCCTTGG | CCAAGAAATTGATTGCCTCTTG | 57 | 156 | SSCP |
| PSY | GTCAAGAACCAGACGCATTG | GCCTTGTCGTCTTACACTTCG | 60 | 250 (700) | SSR |
| RGA1 | GAGTCGTGTACGCTTGGG | CAGTAGGAAAAGGCCCAACA | 64 | 152 | Indel |
| RGA3 | CAATATAAACGCCCGACTGC | TGCTGCTGCTGCTACCTATG | 60 | 150 | SSCP |
| RGA4 | GCAAGCTGCTGATGACTCTG | ACTGGGTTGGGTCAAAGATG | 60 | 193 | SSCP |
| RHB1 | TGGAGATCTTGGGGAATCAG | TGATAGTGGTGCTCGCATTC | 60 | 192 (350) | SSCP |
| Ribo2 | TGGCCTGACTACAATGATGG | GAGCAAGTCCCATGCTTCTC | 60 | 191 (800) | SSCP |
| RIN | TTGCAAAGAGAAGAAATGG | CGTTGTAGGGACTCAAATCG | 60 | 259 | Indel |
| RRP | AAGCTTGGCATTGAGAGGAG | CTGCAGGTCCCACAAACTC | 60 | 370 (700) | SSCP |
| R-Zinc | GCAGGTGTGCACAATTGAAG | TTTCCAAAAGAACCCAATGC | 60 | 300 | SSCP |
| SAGT | GAGAGTGAGAGAGAGAGAGA | AATGACTTGCTGATGAATA | 60 | 171 | Multiallelic |
| SAMDC1 | GCCAATCAGGACCCACATAC | CGTCTTCTGGTGTCACATGG | 60 | 252 | SSCP |
| SAMM | GACCCCGCTCTCTCTCTCTC | GCACCCGTAGATCACCACTT | 60 | 165 | SSCP |
| SDH | ACCTTCAGGCCTTCACACAC | AGAAGACATCCCTCCCTTGC | 60 | 187 | SSCP |
| SeCy | CGAAATTGAGGAGGAGATGG | CAATATCTCCCATGCCAACC | 60 | 214 (330) | SSCP |
| SIP | CAGACGAAGAGATGTACAGATGG | ACAGGATGCTGAGTGGAACC | 60 | 235 | Indel |
| Sod4a | CAAAAGCACTGGAAATGCTG | GGAGTTTTGCGTTCAGTTCC | 60 | 152 (600) | SSCP |
| SPP | CCACTCATTCGTGGGATTTC | TGTTGCAAGTTGAAGCATTG | 60 | 219 | SSCP |
| SPS | CACCAATCTCCAACACATTACC | CTGTTCACCCAGTCGTTTCC | 60 | 158 | SSR |
| ST | GATATCAGCCATGGCACCTC | CGATGATTCCAGTGATGACG | 60 | 197 (900) | Indel |
| ST1 | ATGAGTGGAGATGGGAAAGC | GATTAAGCCCCTCCTTCAGC | 60 | 213 (1500) | Indel |
| Stress | TCTTCCACCACCACAAGGAT | TTGACTCGTGCTTCTCATGC | 60 | 700 (390) | Indel |
| TAPG | CCACCACTCCTCACTCCTTC | GCTTTAGCCCATGCAGAGAG | 60 | 237 | SSR |
| TAT | TGCTCCTACTGTTGGCCTTC | TGCAGGAGATCAAAATGTCG | 60 | 246 (1000) | SSCP |
| Thioest | TCCCGGTGAACCAGTTTTAG | GCAATCTGGGCAACTGCTAC | 60 | 151 | SSCP |
| TIP | CCGTCAGTGCGAACATCTC | GGAGAGAGCAAATGCTGAGG | 60 | 188 (300) | SSCP |
| TP1 | ATTGTGGTTCTGGCCAAGTC | GTTAATATCGGCAGGGCAAG | 60 | 200 | SSCP |
| TTG1 | AGCCTGATACCCCTTTGC | GCAGCTCCCAAATAAGTGC | 60 | 234 | SSCP |
| UFGT | GCTTTGAGCCAAGGTGATTC | TCCAACGACACGTCTTTCTTC | 60 | 208 | Indel/SSCP |
| Unk1 | AGTTCAACCCATACCCTTTGT | GGGCTAAGGGGTTTGCTTAT | 60 | 246 | SSCP |
| Unk2 | TCCTATTCTGTTCCCCGAGA | TTTGCCAAGCATAATTGGTT | 60 | 190 | SSCP |
| Unk3 | GCTGCAGGAATTTGTGGTTT | TTGGGAGAATCATAGCATTTG | 60 | 246 | Indel |
| Unk5 | TGAATGAAATGTGGCGACTC | ATCCATCCTGATCCAACAGC | 60 | 295 (410) | SSCP |
| Unk6 | TCCTACCCACATCCCATCAG | TTCAAAACAACCTGCAAATG | 60 | 225 | SSCP |
| Unk7 | AGGGCAAGCCTACATCTTCC | CCTCTGCACTTTCTCCTTCG | 60 | 232 (340) | Indel/SSCP |
| Unk8 | AAGAAATTTGGTGCCGTCTG | ATCAAGTTGGCCACTTTTGC | 60 | 258 | SSCP |
| Unk9 | CTTTCAATCCGCCAAGAATG | TCCCAAGTGATGGTGTTGTG | 60 | 245 | Indel |
| Unk10 | TAAACAGCCCCAAGAAATGG | CCTTAACCCCTCATCTGTGC | 60 | 206 (406) | SSCP |
| Unk13 | TACCTGCCCAACTGCTATCC | GATGGATGCAACTGGTTTCC | 60 | 223 | SSCP |
| Unk15 | CAAGAAACCATGGGGATCAG | ATCTCACAGGCACTGCAAGC | 60 | 193 | Indel |
| Unk17 | GAGCATGGATTTCTGGCAAC | TCCTCTTCTCCTGCATCACC | 60 | 275 (390) | SSCP |
| Unk18 | CACCATTTTGGAGGATTTCG | TGCTGCAATTGAACAAGCAG | 60 | 232 | SSCP |
| Unk19 | AGGAGTTGCAGAATGGCAAG | ACCAAGATGACCAGCAAAGG | 60 | 243 | SSCP |
| Unk20 | GGAAAACAGCAGGAAAGCAG | CGGTATTTAGCAGCGGTCTC | 60 | 201 (380) | SSCP |
| Unk21 | GCCACTGCAAAAGCCTATTC | AAGGACGATATTTGCTCGGTAG | 57 | 238 | SSR |
| Unk22 | TTGGCGAAGTGTATCAGCAG | AGGTGCTGCAAGGAATTAGC | 60 | 222 | SSCP |
| Unk23 | GACCATCTCCCTGATGATCG | CACAGTCACAATGTCCACCAG | 60 | 250 | SSCP |
| Unk24 | ATAAGCATTTTGGGGTGTGC | GCAATGTGCAGAGACACCAG | 60 | 242 (650) | SSCP |
| Unk25 | GATGATGGAGGGATTGTTGG | GTGAACTGGGTGTCGCATC | 60 | 234 | Indel |
| Unk27 | ACTCTTTGGTGCGGTTCTTG | TTCGTCGCAGGACTATGTTC | 60 | 249 (620) | SSCP |
| Unk28 | CAATGCTGTTGATTGGTTGG | TGCCAAGGCTTAGACATTCC | 60 | 248 (450) | SSCP |
| Unk29 | TGGGATAGACCTGGCAACTC | AGGCCTCTCTTTGAGCTTTG | 60 | 250 (600) | Indel/SSCP |
| Unk30 | AAAGGCCCATCATCATTCAG | AAGGACGATCAAAGGTCACG | 60 | 243 (1000) | SSCP |
| ZXE2 | GTTCATGCTATGCAGCCAAG | AAGCCATTAATGCAGCCATC | 64 | 216 | SSCP |

**^a^**: Expected amplicon size (base pairs); observed size in parenthesis when different from expected.

**^b^**: Type of polymorphism; indel = insertion/deletion, SSR = simple sequence repeats, SSCP = Single Strand Conformational Polymorphism, NP = not polymorphic.
